# Streamwise-travelling viscous waves in channel flows

**DOI:** 10.1007/s10665-018-9953-y

**Published:** 2018-02-23

**Authors:** Pierre Ricco, Peter D. Hicks

**Affiliations:** 1 Department of Mechanical Engineering, University of Sheffield, Sheffield, S1 3JD UK; 2 School of Engineering, Fraser Noble Building, King’s College, University of Aberdeen, Aberdeen, AB24 3UE UK

**Keywords:** Biosensors, Electro-osmosis, Electro-osmotic waves, Love waves, Microfluidics, Mixing, Shear-horizontal surface acoustic waves, Turbulent drag reduction

## Abstract

The unsteady viscous flow induced by streamwise-travelling waves of spanwise wall velocity in an incompressible laminar channel flow is investigated. Wall waves belonging to this category have found important practical applications, such as microfluidic flow manipulation via electro-osmosis and surface acoustic forcing and reduction of wall friction in turbulent wall-bounded flows. An analytical solution composed of the classical streamwise Poiseuille flow and a spanwise velocity profile described by the parabolic cylinder function is found. The solution depends on the bulk Reynolds number *R*, the scaled streamwise wavelength $\lambda$, and the scaled wave phase speed *U*. Numerical solutions are discussed for various combinations of these parameters. The flow is studied by the boundary-layer theory, thereby revealing the dominant physical balances and quantifying the thickness of the near-wall spanwise flow. The Wentzel–Kramers–Brillouin–Jeffreys (WKBJ) theory is also employed to obtain an analytical solution, which is valid across the whole channel. For positive wave speeds which are smaller than or equal to the maximum streamwise velocity, a turning-point behaviour emerges through the WKBJ analysis. Between the wall and the turning point, the wall-normal viscous effects are balanced solely by the convection driven by the wall forcing, while between the turning point and the centreline, the Poiseuille convection balances the wall-normal diffusion. At the turning point, the Poiseuille convection and the convection from the wall forcing cancel each other out, which leads to a constant viscous stress and to the break down of the WKBJ solution. This flow regime is analysed through a WKBJ composite expansion and the Langer method. The Langer solution is simpler and more accurate than the WKBJ composite solution, while the latter quantifies the thickness of the turning-point region. We also discuss how these waves can be generated via surface acoustic forcing and electro-osmosis and propose their use as microfluidic flow mixing devices. For the electro-osmosis case, the Helmholtz–Smoluchowski velocity at the edge of the Debye–Hückel layer, which drives the bulk electrically neutral flow, is obtained by matched asymptotic expansion.

## Introduction

In this paper, we study the unsteady laminar flow generated in a Poiseuille flow channel by the following wall waves of sinusoidal spanwise velocity travelling along the streamwise direction:

1 $$\begin{aligned} w_\mathrm{w}^* = A^* \cos \left[ 2 \pi (x^* - U^* t^*)/\lambda ^*\right] , \end{aligned}$$

where $w_\mathrm{w}^*$ is the spanwise wall velocity, $x^*$ indicates the streamwise direction, $t^*$ denotes time, and $A^*$ denotes the oscillation amplitude. Two parameters define the wall motion: the streamwise wavelength $\lambda ^*$ and the phase speed $U^*$. The third parameter defining the physical system is the Reynolds number *R* based on the bulk velocity of the streamwise Poiseuille flow $U_\mathrm{b}^*$ and the half channel height $h^*$. Our study is theoretical and numerical and aims at a complete characterization of the viscous flow in the parameter space. In the following, a variety of flow configurations where these waves may play an important role are discussed, including flow mixing in microfluidic systems and turbulent drag reduction.

### Travelling waves in microfluidic systems

Small-scale oscillating flows often feature in microfluidic and micro-electromechanical systems. A benefit of the oscillations is the promotion of mixing in the flow, which, as a result of the small length scales and the small velocities involved, is essentially laminar. At such Reynolds numbers, $R \approx 0.1$–10, oscillating flows such as that induced by the wall motion (1) can be enforced by surface acoustic waves or by electro-osmosis waves.

Surface acoustic waves (SAWs) have been utilized extensively for a wide range of microfluidic applications, such as micromixing, micropumping, drop transport, cell handling, and microejectors [1–5], although shear-horizontal waves have never been employed in microfluidic flow mixing. SAWs are created by piezoelectric transduction within a thin solid substrate below a fluid, so that electric power causes the mechanical deformation of the substrate, which, in turn, leads to the motion of the fluid.

Wall-normal Rayleigh acoustic waves have been used for mixing of microfluidic flows [6–9]. However, they generate compression waves in a liquid and suffer from energy dissipation (leaky waves) [6, 10]. When instead in-plane motion occurs, thanks to the mismatch of the sound speeds and densities of the substrate material and the fluid, the acoustic propagation is confined within the substrate, while the fluid flow is incompressible. This is a relevant simplification for the analysis of SAWs because the incompressible Navier–Stokes equations with an imposed slip velocity describe the dynamics (i.e. an analytical solution for streamwise standing waves is found on p. 133 of the book by Bruus [11]). Tan et al. [4], in their Fig. 2c, show interfacial standing shear waves which are in-plane and sinusoidal as an interesting variant of SAWs. Although no bulk flow is present in the system studied by Neumann et al. [12], this example shows that it is possible to generate shear-horizontal acoustic waves in a thin solid substrate to affect an overlying liquid layer.

**Fig. 1:**
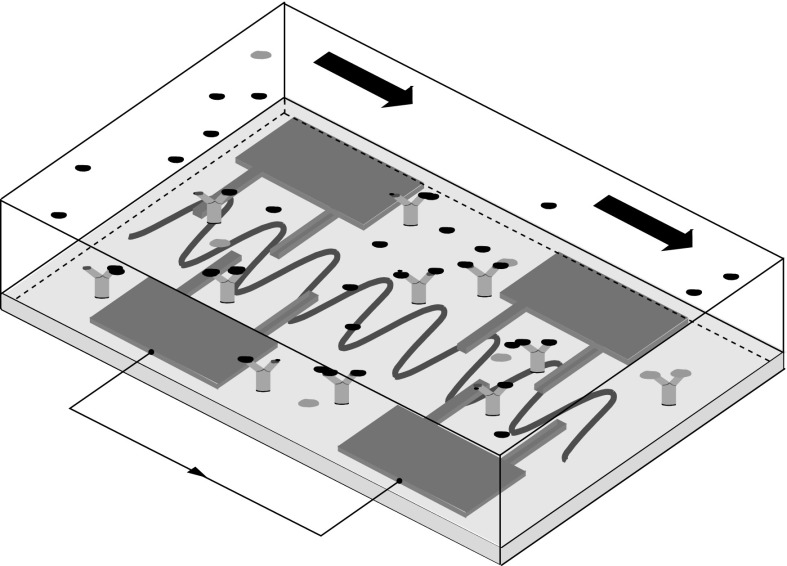
Schematic of a shear-horizontal SAW biosensor, adapted from Lange et al. [13]. The large black arrows indicate the liquid sample flowing over the piezoelectric crystal substrate, the small black arrow denotes the electric signal exchanged between the two interdigital transducers, and the blue sinusoidal line shows the travelling surface acoustic wave

Shear-horizontal surface waves, also called Love waves when a layer of lower acoustic velocity is used for increased sensitivity, have also been studied extensively as efficient biosensors and chemical sensors for flowing solutions because of their low dissipation when compared with wall-normal Rayleigh waves [13, 14]. However, these studies are mainly experimental and Lange et al. [13] indeed remark that improved design of these biosensors can be achieved by studying the fluid dynamics generated by the interaction of the spanwise waves and the overriding streamwise flow. Figure 1 depicts a schematic of a biosensor based on shear-horizontal travelling waves, an excellent technological application of the waves studied herein. Shear-horizontal SAWs waves have also been employed by Neumann et al. [12] to manipulate proteins attached to supported lipid bilayers. Love waves have also been used more recently as non-intrusive rheometers. In particular, the interaction between the SAWs and the liquid has been studied to extract the relationship between the wave attenuation and the viscosity [15, 16].

SAWs are usually characterized by maximum surface velocities of 1 m/s and frequencies in the broad range of 100 kHz–100 MHz. The characteristic wavelength can be up to $100~\upmu \hbox {m}$, i.e. comparable with the microfluidic channel height. Therefore, for Reynolds numbers of the order of unity, realistic ratios between the streamwise wavelength and the channel height can be in the range 0.1–1. The ratio between the wall wave speed and the bulk fluid velocity can vary greatly, namely from null to the order of $10^4$. The latter scenario is more common as it corresponds to a single acoustic wave, travelling at the sound speed of about 1500 m/s below a liquid flowing at about $100~\upmu \hbox {m}/\hbox {s}$. Nonetheless, standing acoustic waves can be generated by the interference of travelling waves [8].

In addition to mechanical wall motions, travelling wave on the walls of a microchannel of the form (1) can also be engendered via electro-osmosis [17–19]. Surface electrodes driven by AC currents below a fluid can generate a uniform plug flow within a very thin charged Debye–Hückel layer, which drags the overlying uncharged fluid by shear stresses. As remarked by Ajdari [18], since these layers are usually much thinner than the radii of curvature of the surface and the channel height, it can be assumed that the uncharged fluid is simply affected by an imposed effective slip velocity, which is linearly related to the electric field, and that the bulk flow is described by the incompressible Navier–Stokes equations. A concise explanation of the physics of wall-based electro-osmosis in microchannels is found in Chang and Yang [20]. These shear motions have also been proposed as an electro-osmotic pumping device to drive fluid along a channel [21, 22]. Micromixing has also been successfully achieved through electro-osmotic wall forcing [23–27].

In line with these microfluidic mixing applications, we complement our theoretical/numerical results with ideas on the laboratory realization of the waves engendered by (1) through surface acoustic forcing by piezoelectric crystals and through electro-osmosis actuation for microfluidic flow mixing (refer to Sect. 8).

### Travelling waves for turbulent drag reduction

The wall wave motion given by (1) has also been studied beneath wall-bounded turbulent flows, first via direct numerical simulations in a turbulent channel flow by Quadrio et al. [31] and Quadrio and Ricco [32], and experimentally in a pipe with rotating sections by Auteri et al. [28] and in a wind-tunnel flow over a deformable Kagome lattice surface by Bird et al. [29, 30]. Drag reduction or drag increase occurs depending on the forcing parameters $\lambda$ and *U*. For the pipe with rotating sections [28] and the wind-tunnel flow over the Kagome surface [29, 30], the bulk Reynolds number is obviously much larger than unity and $\lambda$ is either comparable or a few times larger than the pipe radius in the case of Auteri et al. [28] or the boundary-layer thickness in the case of Bird et al. [29, 30].

It is obviously very different to investigate the flow engendered by the waves given by (1) in the laminar regime or in the fully developed turbulent regime. Nevertheless, there is ample evidence that the laminar profiles generated by spanwise wall motion are very useful to study various aspects of the corresponding fully turbulent flow. Choi et al. [33] and Quadrio and Ricco [32] have indeed verified that the unsteady space-averaged spanwise profile may closely match the corresponding laminar solution. The good agreement occurs when the wall forcing acts on a time scale which is much shorter than the life time of the near-wall turbulent structures. Under these conditions, the drag reduction scales with the thickness of the spanwise boundary layer, which is computed through the laminar solution. Furthermore, the near-wall laminar solutions have been instrumental for the accurate computation of the power spent for moving the wall against the viscous flow resistance, the optimal layer thickness which leads to maximum drag reduction, or the smallest period of wall forcing which guarantees drag reduction [32]. Choiet al. [33] and Ricco et al. [34] have also utilized the laminar Stokes layer solution to define a scaling parameter for drag reduction prediction and Choi [35] has taken advantage of the spanwise laminar flow behaviour to interpret the changes of the near-wall turbulent structures.

### Objectives and structure of the paper

Motivated by the possibility of microfluidic flow manipulation offered by shear-horizontal waves, by their extensive use as bio- and chemical sensors, and by the importance of the laminar solutions for the study of turbulent drag reduction by spanwise forcing, a complete study on the laminar spanwise flow engendered by the wall motion given by (1) is presented herein. The investigation is based on numerical calculations and on asymptotic analysis. The spanwise momentum equation is first simplified to a second-order ordinary differential equation and solved numerically by a second-order finite-difference scheme. Its solution is also expressed analytically through the parabolic cylinder function (hereinafter referred to as PCF).

The Reynolds number *R*, the wave speed *U*, and the wavelength $\lambda$ are treated as asymptotic parameters, thus deriving asymptotic analytical solutions utilizing the boundary-layer and the Wentzel–Kramers–Brillouin–Jeffreys (WKBJ) theories. Employing the boundary-layer scaling, the thicknesses of the near-wall viscous layers are quantified, while the WKBJ solution gives the correct flow structure across the whole channel. When the streamwise diffusion is negligible, the convection term due to the wave motion may balance the streamwise Poiseuille flow convection term in a specified range of wave phase speeds. For these cases, alternative solutions found using WKBJ turning-point composite expansions and the method of Langer [36, 37] are derived. All the asymptotic solutions show excellent agreement with the numerical solutions and offer the further advantage over the numerical approach that the dominant physical balances are revealed. The final aim of our work is to discuss how these waves can be generated in a laboratory via electro-osmosis and surface acoustic forcing, and to propose them as microfluidic flow mixers.

The scaling and the simplification of the spanwise momentum equation are discussed in Sect. 2, while Sect. 3 presents the analytical solution in terms of the PCF. The numerical results are shown in Sect. 4, the boundary-layer theory results are discussed in Sect. 5, while Sect. 6 presents the WKBJ results and Sect. 7 contains the Langer solution. A discussion on future applications of the travelling waves for flow mixing is contained in Sect. 8 and a summary is found in Sect. 9.

## Governing equation

**Fig. 2:**
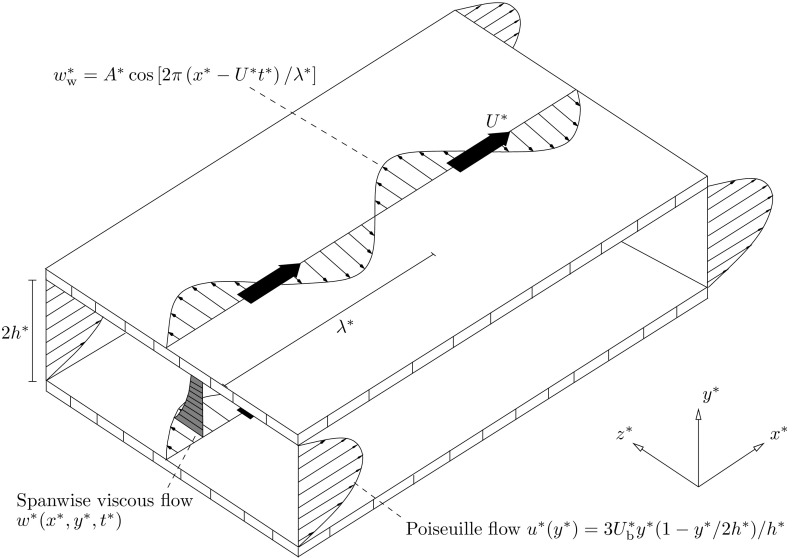
Physical domain for Poiseuille flow with forward-travelling wall waves. The channel width is $2h^*$, $\lambda ^*$ is the streamwise wavelength of the wall forcing, and $U^*$ is the phase speed

Laminar flow confined between two infinite parallel plates at a distance $2h^*$ is considered. The superscript $*$ hereinafter denotes a dimensional quantity. The streamwise, wall-normal, and spanwise directions are indicated by $x^*$, $y^*$, and $z^*$, respectively, while $t^*$ denotes time. The walls move along the spanwise direction with velocity given by (1), where without loss of generality, the wavelength $\lambda ^* > 0$ because the flow is invariant to a change of $\lambda ^*$ to $-\lambda ^*$. The flow is governed by the incompressible Navier–Stokes equations:

2a $$\begin{aligned}&\nabla \cdot \mathbf {u}^* = 0, \end{aligned}$$

2b $$\begin{aligned}&\frac{\partial \mathbf {u}^*}{\partial t^*} + \left( \mathbf {u}^* \cdot \nabla \right) \mathbf {u}^* = -\frac{1}{\rho ^*} \nabla p^* + \nu ^* \nabla ^2 \mathbf {u}^*, \end{aligned}$$

where $\mathbf{u}^*=\{u^*,\,v^*,\,w^*\}$ is the velocity vector with components along $x^*$, $y^*$, and $z^*$, $p^*$ is the pressure, $\rho ^*$ is the density of the fluid, $\nu ^*$ is the kinematic viscosity of the fluid, and $\nabla =\{\partial /\partial x^*,\,\partial /\partial y^*,\,\partial /\partial z^*\}$. At the walls $y^*=0$ and $y^*=2h^*$, the no-slip and no-penetration boundary conditions are

3 $$\begin{aligned} u^*=v^*=0, \quad \text{ and } \quad w^* = A^* {\mathfrak {R}}\big [ \mathrm {e}^{2 \pi \mathrm{i} \left( x^* - U^* t^* \right) /\lambda ^* }\big ], \end{aligned}$$

where $\mathfrak {R}$ indicates the real part. The phase speed $U^*$ can be positive (forward-travelling wave), null (standing wave), or negative (backward-travelling wave). Figure 2 shows the flow domain for forward-travelling waves.

As the boundary conditions (3) depend only on $x^*$ and $t^*$ and a pressure gradient is present only along $x^*$, all terms containing $z^*$ derivatives vanish. The continuity equation (2a) and the *x*-momentum equation (2b) are thus independent of $w^*$. The streamwise flow is the classical Poiseuille flow, while $w^*$ satisfies the simplified *z*-momentum equation

4 $$\begin{aligned} \frac{\partial w^*}{\partial t^*} + u^* \frac{\partial w^*}{\partial x^*} = \nu ^* \left( \frac{\partial ^2 w^*}{\partial x^{*2}} + \frac{\partial ^2 w^*}{\partial y^{*2}} \right) . \end{aligned}$$

The $x^*$ coordinate is now scaled by the wall streamwise wavelength $\lambda ^*$, while the $y^*$ coordinate is scaled by $h^*$ to enable the definition of the dimensionless coordinates $x=x^*/\lambda ^*=O\,(1)$ and $y=y^*/h^*=O\,(1)$. The non-dimensional streamwise velocity $u = u^*/U_\mathrm{b}^* = O\,(1)$ is defined using the bulk velocity $U_\mathrm{b}^* = (1/h^*)\int _0^{h^*} u^*\left( y^*\right) \,\,\mathrm {d}y^*$, while the spanwise velocity $w^*$ is scaled by the wave amplitude $A^*$, i.e. $w = w^*/A^* = O\,(1)$. The time is non-dimensionalized by the period of the wall motion, i.e. $t = t^* U^*/ \lambda ^* = O\,(1)$. In terms of these non-dimensional quantities Eq. (4) becomes

5 $$\begin{aligned} \frac{\partial w}{\partial t} + \frac{U_\mathrm{b}^*}{U^*} u \frac{\partial w}{\partial x} = \frac{\nu ^*}{\lambda ^* U^*} \frac{\partial ^2 w}{\partial x^2} + \frac{\nu ^* \lambda ^*}{h^{*2} U^*} \frac{\partial ^2 w}{\partial y^2}, \end{aligned}$$

subject to $w(0)=w(2)=\mathfrak {R}\{\exp [2\pi \mathrm{i} \left( x-t\right) ]\}$. By introducing the new variable $\xi = x-t$ and by expressing the solution as

6 $$\begin{aligned} w=\mathfrak {R}\Big [W(y)\mathrm {e}^{2\pi \mathrm{i} \xi }\Big ], \end{aligned}$$

Eq. (5) simplifies to

7 $$\begin{aligned} \underbrace{W^{\prime \prime }(y)}_{y\tiny {\text{-diffusion }}} + \Biggl ( \underbrace{\frac{3 \pi \mathrm{i} R}{\lambda } y^2 - \frac{6 \pi \mathrm{i} R}{\lambda } y}_{\tiny {\text{ Poiseuille } \text{ convection }}} \underbrace{+ \frac{2 \pi \mathrm{i} R U}{\lambda }}_{\tiny {\text{ wave } \text{ convection }}} \underbrace{- \frac{4 \pi ^2}{{\lambda }^2}}_{x\tiny {\text{-diffusion }}} \Biggr ) W(y) = 0, \end{aligned}$$

subject to

8 $$\begin{aligned} W(0)=W(2)=1. \end{aligned}$$

A prime indicates differentiation with respect to *y* and use has been made of the Poiseuille solution, $u=3y(1-y/2)$. Three parameters appear in Eq. (7): the Reynolds number $R=U_\mathrm{b}^* h^*/\nu ^*$, the scaled phase speed $U = U^*/U_\mathrm{b}^*$, and the scaled wavelength $\lambda = \lambda ^*/h^*$.

## Analytical solution in terms of the parabolic cylinder function

By introducing the wall-normal coordinate $\hat{y}=\phi (1-y)$, where $\phi = \sqrt{2}[3 \pi R/(\lambda \mathrm{i})]^{1/4}$, and the spanwise velocity $\widehat{W}\left( \hat{y} \right) =W\left( y \right)$, Eq. (7) simplifies to

9 $$\begin{aligned} \widehat{W}^{\prime \prime }\left( \hat{y} \right) +\left( a+\frac{1}{2}-\frac{\hat{y}^2}{4}\right) \widehat{W}\left( \hat{y} \right) =0, \end{aligned}$$

where $a = \sqrt{\mathrm{i} \pi /(3 \lambda R)} \left[ \mathrm{i} R (U-3/2) - 2 \pi /\lambda \right] - 1/2$. The boundary conditions (8) become $\widehat{W}\left( \pm \phi \right) =1$. Equation (9) is in the form of Eq. (3.5.11) on p. 96 in Bender and Orszag [38] and therefore the solution of Eq. (5) can be written in terms of two linearly independent PCFs:

10 $$\begin{aligned} w(x,y,t;R,U,\lambda )=\mathfrak {R}\left\{ \frac{\mathcal {D}_a[\phi (1-y)] + \mathcal {D}_a[\phi (y-1)]}{\mathcal {D}_a(\phi ) + \mathcal {D}_a(-\phi )}\mathrm {e}^{2\pi \mathrm{i} (x-t)}\right\} . \end{aligned}$$

While this expression provides an analytical solution for *w*, its practical value for determining the actual spanwise flow velocity is somewhat limited. This is because, to the best of our knowledge, there does not exist yet a robust numerical code which solves for the PCF in the entire complex plane. Temme [39] offers a complete list of journal articles on the numerical algorithms, outlining the restrictions on the complex argument. He states that “...constructing reliable software for all possible combinations of the complex parameters is a challenging problem”. *Mathematica* solves the complex-to-complex PCF, but it produces erroneous results for spanwise viscous layers extending to the whole channel width. As a full computational implementation of a numerical code for complex-to-complex PCF lies outside of the scope of the present work, numerical and asymptotic methods will now be employed to study the flow.

## Numerical results

Flow profiles are first obtained by solving Eq. (7) numerically through a second-order finite-difference scheme with uniform mesh size along *y* [40]. We have chosen an implicit scheme to avoid stiffness-related problems. As the flow is symmetric with respect to the centreline, only half of the domain along *y* needs to be considered, and the boundary conditions become $W(0)=1, W^\prime (1)=0$. In the following figures, the numerical profiles are shown for eight $\xi$ values, equally spaced across one oscillation period.

**Fig. 3:**
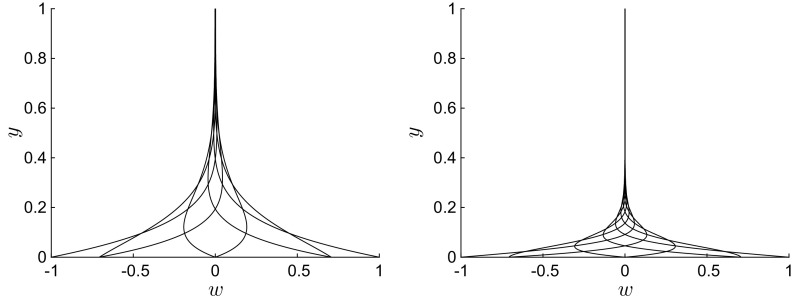
Instantaneous profiles of *w*(*y*) for $U=10$ (left) and $U=100$ (right), $R=1$, $\lambda =1$. Profiles are shown uniformly spaced across one wall oscillation

Increasing the phase speed *U* renders the spanwise viscous region thinner, as shown in Fig. 3 for $R=1$, $\lambda =1$, $U=10$ (left), and $U=100$ (right). This behaviour is the same as for $\lambda /R \ll 1$, where the boundary-layer thickness becomes thinner as the frequency increases (refer to contour plot in Quadrio and Ricco [32, Fig. 8]). In the cases shown in Fig. 3, all four terms in Eq. (4) play a role, as the unsteadiness and the transport due to the Poiseuille flow are balanced by viscous diffusion along *x* and *y*. These profiles are very similar to the classical Stokes problem profiles. Note that, for $\lambda =R=1$ and $U=10$ (and smaller), a boundary layer does not exist as viscous effects diffuse across the whole wall-normal extent of the channel.

**Fig. 4:**
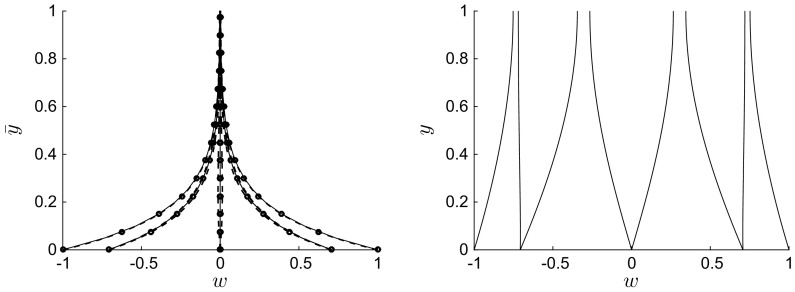
Left: Profiles of $w(\bar{y})$, where $\bar{y}=y/\lambda$, for $R=1$, $U=0$, $\lambda =0.01$ (solid line), $\lambda =0.1$ (circles), $\lambda =1$ (dashed line). Right: Profiles of *w*(*y*) for $R=1$, $U=0$, $\lambda =10$

Flows for $R=1$ with $U=0$ and wavelengths $\lambda =0.01$, 0.1, 1 are shown in Fig. 4(left). A boundary layer emerges as $\lambda$ decreases, and the flow is self-similar as the wall-normal coordinate is rescaled by the streamwise wavelength, $\bar{y}=y/\lambda =O\,(1)$, in the limit $\lambda \ll 1$. The rescaled profiles show excellent agreement, especially near the wall when the wall velocity is large. By introducing $\bar{y}$ in (7) and taking the limit $\lambda \ll 1$, the solution to Eq. (7) is $w=\mathfrak {R}\left\{ \exp \left[ 2\pi \left( \mathrm{i} \xi - y/\lambda \right) \right] \right\}$, as clearly shown in Fig. 4(left). This asymptotic scaling is confirmed by the analysis given in Sect. 5. No convective transport is at work: the physical balance is between the wall-normal and the streamwise viscous diffusion. As the wavelength increases to $\lambda =10$, Fig. 4(right) shows that the velocity tends to a constant value along *y*.

**Fig. 5:**
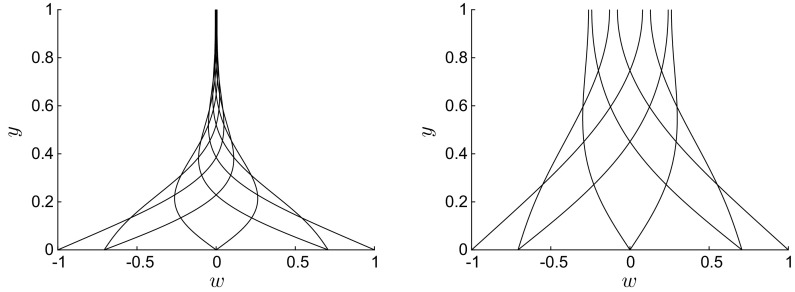
Profiles of *w*(*y*) for $\lambda =100$ (left) and $\lambda =1000$ (right), $R=1000$, $U=0$

**Fig. 6:**
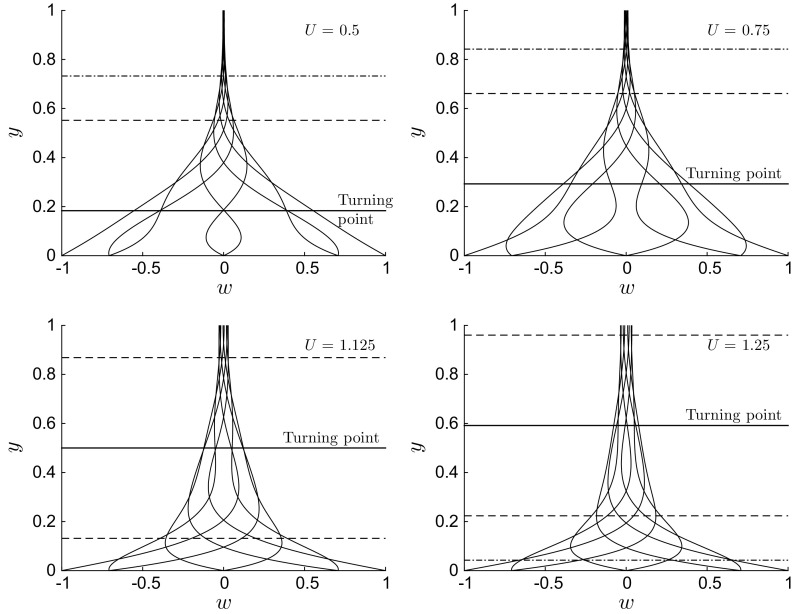
Profiles of *w*(*y*) for $\lambda =50$, $R=1000$ and $U=0.5$ (top left), $U=0.75$ (top right), $U=1.125$ (bottom left), and $U = 1.25$ (bottom right). The horizontal solid lines indicate the location of the turning point $y_0$, while the dashed line $\left( \text{ at }~y-y_0=\pm \left( \lambda /R\right) ^{1/3}\right)$ and dashed-dotted line $\left( \text{ at }~y-y_0=\pm \left( \lambda /R\right) ^{1/5}\right)$ mark the boundaries of the matching region studied in Sect. 6.1

Figure 5 presents profiles for steady-wave flows at high Reynolds number $R=1000$ and with long wavelength (left: $\lambda =100$ and right: $\lambda =1000$). The streamwise viscous diffusion effects, represented by the last term in (7), are negligible. Like the flows at order-one Reynolds number shown in Fig. 3, the spanwise flow occupies the entire channel, with the viscous region becoming thinner as $\lambda /R$ decreases. For the case with $\lambda =1000$ (Fig. 5, right), the spanwise velocity is finite at the centreline.

Profiles for $\lambda \ll R$ resemble the classical Stokes solution for high positive *U* and for negative *U*. Trends similar to the exponentially decaying profiles shown in Fig. 4 are found when $\lambda \ll 1$. However, the profiles vary significantly from the classical Stokes solution when $U \approx 1$ or smaller (and positive) and $\lambda$ is sufficiently larger than 1 and smaller than *R*. Figure 6 shows four sets of profiles for $\lambda =50$, $R=1000$, and $0.5 \le U \le 1.25$. The trends are similar to the profiles of the Stokes layer only in the upper portion of the viscous layer, while at lower locations the trends show oscillatory behaviour which is distinctly different from the Stokes layer. For example, profiles for $U=0.75$ with $w=0.7$ at $y=0$ may decay and change their curvature as *y* increases without crossing the $w=0$ line for the whole channel extent. The spanwise velocity may therefore be positive along the whole channel, which is never the case for the Stokes layer.

Solving the governing equation (7) represents a simple numerical exercise. However, it is clear from the numerical results presented that restricting the analysis to a computational endeavour severely limits the understanding of the physical problem. Therefore, asymptotic methods, i.e. the boundary-layer theory in Sect. 5, the WKBJ theory in Sect. 6, and the Langer theory in Sect. 7, are employed. These analyses are useful because approximate analytical solutions of (7) are obtained and because insight on the physics is gained, which cannot be revealed either through the full numerical solution or the PCF analytical expression (10). In particular, the asymptotic approach quantifies the thickness of the spanwise viscous layer, highlighting the physical balance very near the wall, and explains the occurrence of the wiggly behaviour for wave speeds comparable with the bulk velocity, shown in Fig. 6 (refer to Sect. 6). The theoretical analysis precisely identifies the parameter range for this turning-point regime, i.e. $R^{-1} \ll \lambda \ll R$ and $0 \le U \le 3/2$, and quantifies the thickness of the thin turning-point layer and of the other two order-one regions which confine this layer. The physical balances in these three layers are revealed, which explains the mathematical forms of their asymptotic solutions. The solid lines in Fig. 6, located at $y = 1 - \sqrt{1 - 2U/3}$, indicate the turning-point location. It will be further shown that the asymptotic analysis is also useful for the design of the proposed micromixer based on the travelling waves. The boundary-layer theory indeed identifies the cases where the spanwise flow is confined to a very thin wall-bounded layer, which are clearly not candidates as efficient mixers because the bulk flow, where the mixing is required, is largely unaffected by the wall motion.

## Boundary-layer theory

To expedite the analysis, the asymptotic parameter $\varepsilon \equiv \lambda /R$ is defined, which can be written asAs also clear from multiplying each term of Eq. (5) by $A^*U^*/\lambda ^*$, $\varepsilon$ represents the ratio between the wall-normal viscous effects and the convection effects due to the transport of the Poiseuille flow on the streamwise gradient of the spanwise flow. When $U=O(1)$ these convection effects are also comparable to the unsteadiness due to the wave motion. In terms of $\varepsilon$, Eq. (7) is written as

11 $$\begin{aligned} \varepsilon W^{\prime \prime }\left( y \right) + \left( 3 \pi \mathrm{i} y^2 - 6 \pi \mathrm{i} y + 2 \pi \mathrm{i} U - \frac{4 \pi ^2 \varepsilon }{\lambda ^2}\right) W\left( y \right) = 0. \end{aligned}$$

The cases for $\varepsilon \ll 1$ are studied in Sect. 5.1 and the cases for $\varepsilon =O\,(1)$ and $\varepsilon \gg 1$ are studied in Sect. 5.2.

### The small-$\varepsilon$ regime: $\lambda \ll R$

In the limit $\varepsilon \ll 1$, the solution of Eq. (11) is $W=0$, which satisfies the boundary condition $W^\prime (1)=0$, but not the wall condition $W(0)=1$. A boundary layer therefore exists in the proximity of the wall, where the solution varies rapidly. When $y \ll 1$, the coordinate *y* is rescaled as $Y=y/\delta =O\,(1)$, where  $\delta =\varepsilon ^\beta$ is the boundary-layer thickness and $\beta$ is an unknown positive number. Within the boundary layer, Eq. (11) becomes

12 $$\begin{aligned} \overline{W}^{\prime \prime }\left( Y \right) + \left( 3 \pi \mathrm{i} Y^2 \varepsilon ^{4 \beta - 1} - 6 \pi \mathrm{i} Y \varepsilon ^{3 \beta - 1} + 2 \pi \mathrm{i} U \varepsilon ^{2 \beta - 1} - \frac{4 \pi }{\lambda ^2} \varepsilon ^{2 \beta } \right) \overline{W}\left( Y \right) = 0, \end{aligned}$$

where $\overline{W}(Y)=W(y)$. There are two possible distinguished limits in (12): $\beta =1/2$ and $\beta =1/3$. This scenario is typically encountered in nested boundary-layer problems (refer to Bender and Orszag [38, example 6 on p. 453]), where an inner-inner boundary layer must exist on one side of the domain because the inner boundary layer does not satisfy the boundary condition. However, only one boundary layer exists in our case and the two $\beta$ values correspond to distinguished flow regimes which match asymptotically in the parameter space ($\lambda ,R$). Equation (12) is written as

13a $$\begin{aligned} \beta = 1/2:&\quad \overline{W}^{\prime \prime }\left( Y \right) + \left( 3 \pi \mathrm{i} \varepsilon Y^2 - 6 \pi \mathrm{i} \varepsilon ^{1/2} Y + 2 \pi \mathrm{i} U - \frac{4 \pi ^2 \varepsilon }{\lambda ^2} \right) \overline{W}\left( Y \right) = 0, \end{aligned}$$

13b $$\begin{aligned} \beta = 1/3:&\quad \overline{W}^{\prime \prime }\left( Y \right) + \left( 3 \pi \mathrm{i} \varepsilon ^{1/3} Y^2 - 6 \pi \mathrm{i} Y + \frac{2 \pi \mathrm{i} U}{\varepsilon ^{1/3}} - \frac{4 \pi ^2 \varepsilon ^{2/3}}{\lambda ^2} \right) \overline{W}\left( Y \right) = 0, \end{aligned}$$

subject to $\overline{W}(0)=1$ and $\overline{W}(\infty )=0$.

The case with $\beta =1/2$ is studied first. The terms in (13a) that are proportional to *Y* and $Y^2$ may be neglected when the *y* diffusion balances either the convection term due to the wave transport, i.e. $U=O\,(1)$, or the spanwise viscous diffusion term, i.e. $\lambda R=O\,(1)$, or both. Equation (13a) can then be simplified to

14 $$\begin{aligned} \overline{W}^{\prime \prime }\left( Y \right) + \left( 2 \pi \mathrm{i} U - \frac{4 \pi ^2}{\lambda R} \right) \overline{W}\left( Y \right) = 0. \end{aligned}$$

The solution to Eq. (14) is

15 $$\begin{aligned} W\left( y \right) = \exp \left[ \text{ sgn }(U) \sqrt{\frac{4 \pi ^2}{\lambda ^2} - \frac{2 \pi \mathrm{i} U R}{\lambda }} y \right] , \end{aligned}$$

where the branch cut in the square root is taken as the negative real axis. The convection effects brought about by the travelling wave are balanced by the viscous effects along the *x* and *y* directions, and the boundary-layer thickness is $\delta =O\,(\lambda )= O\left( \sqrt{\lambda /(U R)}\right)$.

When $U \gg (\lambda R)^{-1}$, expression (15) simplifies to

16 $$\begin{aligned} W\left( y \right) = \exp \left[ \text{ sgn }(U) \mathrm{i}^{3/2} \sqrt{\frac{2 \pi U R}{\lambda }} y \right] = \exp \left[ (\mathrm{i}-1) \sqrt{\frac{\omega ^*}{\nu ^*}} y^* \right] , \end{aligned}$$

which identifies the oscillating-wall regime. The viscous effects along *x* are negligible. Expression (16), combined with (6), leads to the solution of the Stokes problem for a flat plate oscillating sinusoidally in time beneath a stationary fluid. This is because in this high-frequency limit the streamwise-dependent term in the exponent in (6) is negligible with respect to the unsteady term in (6). The boundary-layer thickness is $\delta =O\left( \sqrt{\lambda /(U R)}\right)$. This flow was also studied by McHale et al. [41] for a shear-horizontal shear wave travelling below a stationary fluid as an idealized model of a quartz crystal microbalance used, among many applications, for in situ monitoring of film deposition and analysis of polymer coatings.

When $U \ll (\lambda R)^{-1}$, expression (15) simplifies to

17 $$\begin{aligned} W\left( y \right) = \exp \left( - \frac{2 \pi y}{\lambda } \right) , \end{aligned}$$

which identifies the regime where the *x* and *y* viscous diffusion effects balance each other to give a boundary-layer thickness $\delta =O\left( \lambda \right)$ (refer to the numerical solution in Fig. 4(left)). This flow is shown to be steady by rescaling time by the average time taken by a fluid particle to cover a distance $\lambda ^*$ along $x^*$, i.e. $\tilde{t} = t^* U_\mathrm{b}^*/\lambda ^*$. The rescaling is necessary because the wave speed is now small. It follows that the exponent in (6) is $2 \pi \mathrm{i} \left( x - U \tilde{t}\right)$, which simplifies to $2 \pi \mathrm{i} x$ for small *U*.

In Eq. (13a), the term involving $Y^2$ is always negligible with respect to the term involving *Y*. However, if neither of the other two terms multiplying $\overline{W}$ (the convective term containing *U* and the *x*-diffusion term containing $(\lambda R)^{-1}$) is $O\,(1)$ and at least one balances the term $\varepsilon ^{1/2} Y$, there is no term to balance the *y* viscous diffusion term. This may occur when $U=O\bigl (\sqrt{\lambda /R}\bigr )$ and $\lambda =O\,(R^{-1/3})$, when $U=O\bigl (\sqrt{\lambda /R}\bigr )$ and $\lambda \gg R^{-1/3}$, or when $U \ll \sqrt{\lambda /R}$ and $\lambda =O\,(R^{-1/3})$. When $U \ll \sqrt{\lambda /R}$ and $\lambda \gg R^{-1/3}$, the term proportional to *Y* alone balances the *y* viscous diffusion term, leading to the steady-wave case studied by Viotti et al. [42]. The scaling with $\beta =1/2$ therefore breaks down because the term proportional to $\overline{W}$ is always smaller than the wall-normal viscous term and the scaling with $\beta =1/3$ applies.

The $\beta =1/3$ case is now investigated. The solution to (13b) is written in terms of the Airy function of the first kind as

18 $$\begin{aligned} W\left( y \right) = \left\{ \text{ Ai }\left[ \mathrm {e}^{-5\pi \mathrm{i}/6} \left( \frac{2 \pi R}{9 \lambda }\right) ^{1/3} \left( U + \frac{2 \pi \mathrm{i}}{\lambda R} \right) \right] \right\} ^{-1} \text{ Ai }\left[ \left( \frac{2 \pi \mathrm{i} R}{9 \lambda }\right) ^{1/3} \left( 3 y - U - \frac{2 \pi \mathrm{i}}{\lambda R} \right) \right] , \end{aligned}$$

which was found by Quadrio and Ricco [32]. Expression (18) identifies the travelling wave regime with streamwise viscous diffusion and is valid when $U = O\,((\lambda /R)^{1/3})$ and $\lambda = O\,(R^{-1/2})$, i.e. when the convective transport due to the waves and the streamwise viscous diffusion balance the Poiseuille flow convective transport and the wall-normal viscous diffusion. The streamwise viscous diffusion effects, denoted by the last term in (18), may be neglected when $\lambda \gg R^{-1/2}$, namely when the last term in (13b) is negligibly small. When $U \ll (\lambda /R)^{1/3}$ and $\lambda \gg R^{-1/2}$, the steady-wave regime with no *x* diffusion effects, studied by Viotti et al. [42], is retained as the last two terms in the argument of the Airy function in (18) are negligible. The steady-wave regime with streamwise diffusion is found when $U \ll (\lambda /R)^{1/3}$ and $\lambda = O\,(R^{-1/2})$.

For large *U*, the asymptotic limit of (18) has been studied by Quadrio and Ricco [32]: the classical solution (16) to the classical Stokes problem is recovered. Analogously, the thin-layer steady-wave regime solution (17) is found through the asymptotic expansion of Eq. (18) as $\lambda \ll R^{-1/2}$ and  $U=O\,((\lambda R)^{-1})$ or smaller. In this limit, the argument $\zeta$ of the Airy function in Eq. (18) is unbounded and

19 $$\begin{aligned} \zeta \sim \mathrm {e}^{-\pi \mathrm{i}/3}\left( \frac{2 \pi R}{9 \lambda } \right) ^{1/3} \left( 3 \mathrm{i} y - \mathrm{i} U + \frac{2 \pi }{\lambda R} \right) \quad \text{ as } \quad \left| \zeta \right| \rightarrow \infty . \end{aligned}$$

The following asymptotic formula therefore applies:

20 $$\begin{aligned} \text{ Ai }(\zeta ) \sim \frac{\mathrm {e}^{-2 {\zeta }^{3/2}/3}}{2 \sqrt{\pi } {\zeta }^{1/4}} \sum _{k=0}^{\infty } (-1)^k c_k \left( \frac{2 {\zeta }^{3/2}}{3}\right) ^{-k} \quad \text{ as } \quad \left| \zeta \right| \rightarrow \infty , ~~ \left| \text{ arg }(\zeta ) \right| < \pi , \end{aligned}$$

where $c_k$ are given in Abramowitz and Stegun [43, 10.4.59 on p. 448]. By substituting (19) into Eqs. (20) and (18), one finds

21 $$\begin{aligned} W\left( y \right) = \exp {\left\{ \frac{16 \mathrm{i} \pi ^3}{9 \lambda ^2 R (2 \pi - \mathrm{i} U \lambda R)} \left\{ \left[ 1 + \frac{3 \mathrm{i} y \lambda R (2 \pi - \mathrm{i} U \lambda R) }{4 \pi ^2} \right] ^{3/2} - 1 \right\} \right\} }. \end{aligned}$$

Expression (21) reduces to (17) by expressing the algebraic term in (21) through a Taylor series expansion with respect to $\lambda \ll 1$, i.e.

$$\begin{aligned} \left[ 1 + \frac{3 \mathrm{i} y \lambda R (2 \pi - \mathrm{i} U \lambda R)}{4 \pi ^2} \right] ^{3/2}= 1 + \frac{9 \mathrm{i} y \lambda R (2 \pi - \mathrm{i} U \lambda R)}{8 \pi ^2} + O\,(\lambda ^2). \end{aligned}$$

**Fig. 7:**
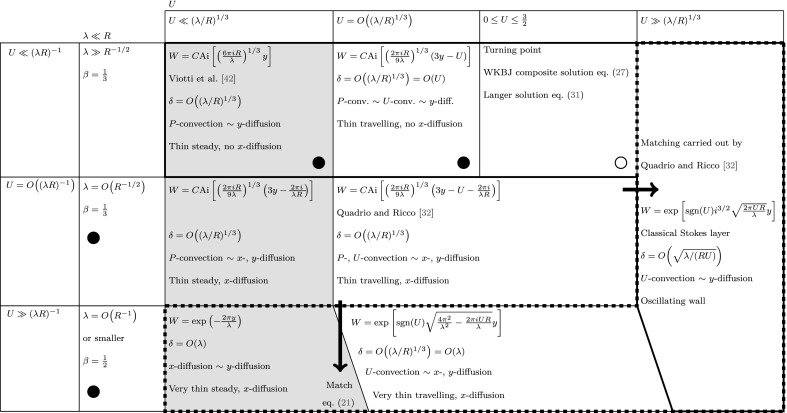
Schematic of asymptotic regimes for the $\lambda \ll R$ case. The quantity *C* is computed in each case by setting $y=0$ in the argument of the Airy function. The black dot indicates cases for which the WKBJ solution without turning point, Eq. (25), is valid, and the white dot indicates cases for which the turning-point WKBJ solution for $0\le U \le 3/2$ and the Langer solution, studied Sects. 6.1 and 7 respectively, are valid. The symbol $\sim$ denotes a physical balance in this figure and in Fig. 8. The convective effects due to the Poiseuille flow and the wall wave are denoted by *P*-convection and *U*-convection, respectively, while the viscous diffusion effects along the streamwise and wall-normal directions are indicated by *x*-diffusion and *y*-diffusion, respectively. The thick solid line contains cases with negligible streamwise viscous diffusion, the thick dashed line contains cases with negligible convective effects related to the Poiseuille flow, and the grey cases are not influenced by convective effects induced by the wall motion. Note that the lines distinguishing the bottom cases are oblique because the regime for $U \ll (\lambda R)^{-1}$ expands as $\lambda$ becomes smaller

It is thus shown that the asymptotic solutions corresponding to the two distinguished limits of (12), for $\beta =1/2$ and $\beta =1/3$, match asymptotically in the parameter space. Figure 7 shows a schematic of the asymptotic regimes for $\lambda \ll R$.

**Fig. 8:**
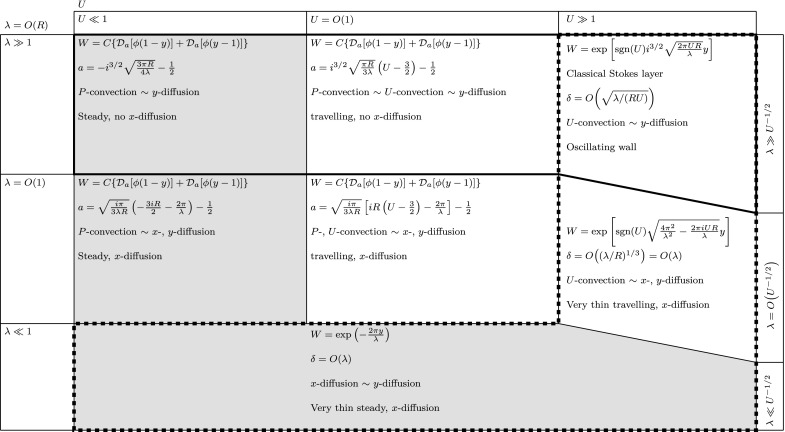
Schematic of asymptotic regimes for $\lambda = O\,(R)$, where $C=[\mathcal {D}_a(\phi ) + \mathcal {D}_a(-\phi )]^{-1}$. The same legend of Fig. 7 applies

### The order-one-$\varepsilon$ and large-$\varepsilon$ regimes: $\lambda = O\,(R)$ and $\lambda \gg R$

The full PCF solution (10) applies when $\lambda = O\,(R) = O\,(1)$ and $U = O\,(1)$. When the streamwise diffusion is negligible, i.e. for $\lambda \gg 1$ and $U = O\,(1)$, the PCF solution (10) remains valid, albeit with $a = \mathrm{i}^{3/2} \sqrt{\pi R/(3 \lambda )} (U-3/2) - 1/2$. These two solutions are still valid when $U \ll 1$, with $a = - \sqrt{\mathrm{i}\pi /(3 \lambda R)} \left[ 3 \mathrm{i} R/2 + 2 \pi /\lambda \right] - 1/2$ when $\lambda = O\,(1)$, and $a = - \mathrm{i}^{3/2}\sqrt{3 \pi R/(4 \lambda )}-1/2$ when $\lambda \gg 1$. When the streamwise diffusion effects dominate, i.e. when $\lambda = O\,(R) \ll 1$ and $U = O\,(1)$ or smaller, Eq. (7) simplifies as only the last term in parenthesis is retained. Hence the solution is given by Eq. (17).

When $U \gg 1$ and $\lambda =O\,(U^{-1/2})$, Eq. (7) can be simplified in similar fashion to the $\lambda \ll R$ regime. It reduces to

22 $$\begin{aligned} W^{\prime \prime }\left( y \right) + \left( \frac{2 \pi \mathrm{i} U R}{\lambda } - \frac{4 \pi ^2}{\lambda ^2} \right) W\left( y \right) = 0, \end{aligned}$$

whose solution is (15). The two simplified solutions, i.e. (16) and (17), are obtained in the limits $\lambda \gg U^{-1/2}$ and $\lambda \ll U^{-1/2}$, respectively. Figure 8 shows a schematic of the asymptotic regimes for $\lambda =O\,(R)$. Note that a boundary layer exists only when the convection due to the wall motion is negligible (areas enclosed by dashed line), whereas the viscous effects extend throughout the entire channel when the PCF solutions apply. The trapezoid in the top right corner of Fig. 8 indicates that the classical Stokes layer occurs when $\lambda \gg U^{-1/2}$, and hence, for every value of *U*, there exists a very large $\lambda$ above which the classical Stokes layer is always recovered.

When $\lambda \gg R$, Eq. (7) simplifies to Eq. (22). The solutions are (15), (16), and (17) when $U=O\,((\lambda R)^{-1})$, $U \gg (\lambda R)^{-1}$, and $U \ll (\lambda R)^{-1}$, respectively. We close this section by pointing out that the boundary-layer analysis reveals no information on the physics behind the peculiar profiles shown in Fig. 6. This is studied through the WKBJ theory in Sect. 6.

## WKBJ theory

An asymptotic solution to (11) is found by the WKBJ physical-optics theory when $\lambda \ll R$ ($\varepsilon \ll 1$). Cases for which $\lambda =O\,(R^{-1})$ are first considered. The solution is [38]

23 $$\begin{aligned} W(y) = \frac{P_0^{1/4}( 1 - \mathrm {e}^{-\psi })}{2 P\left( y \right) ^{1/4} \sinh \psi } \exp \left[ \sqrt{\frac{\mathrm{i}}{\varepsilon }} \int _0^y \sqrt{P\left( \check{y} \right) } \,\,\mathrm {d}\check{y} \right] + \frac{P_0^{1/4} (\mathrm {e}^{\psi }-1)}{2 P\left( y \right) ^{1/4} \sinh \psi } \exp \left[ -\sqrt{\frac{\mathrm{i}}{\varepsilon }} \int _0^y \sqrt{P\left( \check{y} \right) } \,\,\mathrm {d}\check{y} \right] , \end{aligned}$$

where $P\left( y \right) =-3 \pi y^2 + 6 \pi y - 2 \pi U - 4 \mathrm{i} \pi ^2/(\lambda R)$, $P_0=P\left( 0 \right) =P\left( 2 \right) =- 2 \pi U - 4 \mathrm{i} \pi ^2/\lambda R$, and $\psi = \sqrt{\mathrm{i}/\varepsilon } \int _0^2 \sqrt{P\left( \check{y} \right) } \,\,\mathrm {d}\check{y}$. It is easy to confirm that both conditions of validity discussed by Bender and Orszag [38, pp. 493–494] are verified. As

24 $$\begin{aligned} \int _0^y \sqrt{P\left( \check{y} \right) } \,\,\mathrm {d}\check{y} = \frac{\sqrt{3 \pi } \mathrm{i}}{2} \left[ \left( 1-y\right) \alpha \left( y \right) + \sqrt{b} + \left( 1-b\right) \ln \left[ \frac{y - 1 + \alpha \left( y \right) }{\sqrt{b} - 1}\right] \right] , \end{aligned}$$

where $\alpha \left( y \right) = \sqrt{y^2 - 2 y + b}$ and $b = 4 \pi \mathrm{i}/(3 \lambda R) + 2 U/3$, the solution (23) becomes

25 $$\begin{aligned} W(y)= & {} \frac{P_0^{1/4} (1 - \mathrm {e}^{-\psi })}{2 P\left( y \right) ^{1/4} \sinh \psi } \left[ \frac{y-1+\alpha \left( y \right) }{\sqrt{b}-1}\right] ^{\gamma \left( 1-b\right) } \exp \left[ \alpha \left( y \right) \gamma \left( 1-y\right) - \gamma \sqrt{b} \right] + \nonumber \\&\, \frac{P_0^{1/4} \left( \mathrm {e}^{\psi }-1\right) }{2 P\left( y \right) ^{1/4} \sinh \psi } \left[ \frac{\sqrt{b} - 1}{y-1+\alpha \left( y \right) }\right] ^{\gamma \left( 1-b\right) } \exp \left[ \alpha \left( y \right) \gamma \left( y-1\right) + \gamma \sqrt{b} \right] , \end{aligned}$$

where $$\gamma = \sqrt{3 \pi R} \mathrm{i}^{3/2}/\left( 2 \sqrt{\lambda }\right) $$ and $$\psi = \gamma (1 - b) \ln \left[ \left( \sqrt{b} + 1\right) /\left( \sqrt{b} - 1\right) \right] - 2 \gamma \sqrt{b}$$.

**Fig. 9:**
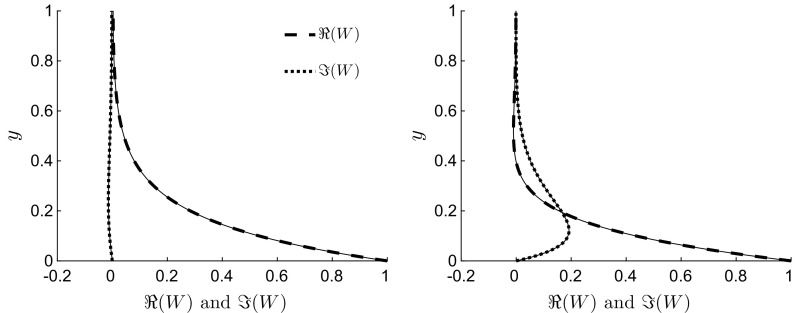
Profiles of the real (dashed) and imaginary (dotted) components of the WKBJ solution (25) for $\lambda =1$, $R=1$ and $U=0$ (left), and $\lambda =1$, $R=1$ and $U=10$ (right) ($\varepsilon =1$ in both cases). The solid profiles are the numerical solutions

Figure 9 shows the profiles of the real and imaginary parts of $W\left( y \right)$ for $\lambda =1$, $R=1$ and $U=0$ (left) and $\lambda =1$, $R=1$ and $U=10$ (right) given by (25). Both the real (dashed) and imaginary (dotted) theoretical profiles are compared to the numerical profiles (thin solid lines), with excellent agreement obtained, although $\varepsilon = \lambda /R = 1$ in both cases. The corresponding spanwise velocities $w=\mathfrak {R}\,\left[ W\left( y \right) \exp \;\left( 2 \pi \mathrm{i} \xi \right) \right]$ for these cases are shown in Figs. 4(left) and 3(left), respectively.

### Turning-point solution by matched asymptotic expansion

In Eq. (11), the streamwise diffusion is negligible when the last term in the parenthesis is smaller than all the other terms. When $U=O(1)$, this occurs when $\lambda \gg R^{-1/2}$. Equation (11) simplifies to

26 $$\begin{aligned} \varepsilon W^{\prime \prime }\left( y \right) - \mathrm{i} \overline{P}\left( y \right) W\left( y \right) = 0, \end{aligned}$$

where $\overline{P}\left( y \right) = -3 \pi y^2 + 6 \pi y - 2 \pi U$. In this limit, the WKBJ asymptotic solution (25) is not valid for $0 \le U \le 3/2$ because $\overline{P}\left( y \right) = 0$ when $y = y_0 = 1 - \sqrt{1 - 2U/3}$, which causes the WKBJ solution (25) to become unbounded there. In this case, a turning point occurs at $y=y_0$. The WKBJ theory reveals that the profiles shown in Fig. 6 belong to this category and is able to define precisely the parameters for which this behaviour occurs. For $0 < U \ll 1$, the turning point is located at $y_0 \sim U/3$. The relationship between $y_0$ and *U* is shown in Fig. 12(left) of Appendix C.

Following Bender and Orszag [38], a composite WKBJ expansion can be constructed in the half channel $0\le y \le 1$, i.e.

27 $$\begin{aligned} W\left( y \right) = W_\mathrm{w}\left( y \right) + W_0\left( y \right) + W_\mathrm{m}\left( y \right) - W_{0\mathrm{w}}^\mathrm{c}\left( y \right) - W_{0\mathrm{m}}^\mathrm{c}\left( y \right) , \end{aligned}$$

where

28a $$\begin{aligned} W_\mathrm{m}= & {} \frac{a_\mathrm{m}}{\left[ \mathrm{i} \overline{P}\left( y \right) \right] ^{1/4}} \exp \,\left[ -\sqrt{\frac{\mathrm{i}}{\varepsilon }} \kappa \left( y \right) \right] + \frac{b_\mathrm{m}}{\left[ \mathrm{i} \overline{P}\left( y \right) \right] ^{1/4}} \exp \,\left[ \sqrt{\frac{\mathrm{i}}{\varepsilon }} \kappa \left( y \right) \right] , \end{aligned}$$

28b $$\begin{aligned} W_0= & {} a_0 \text{ Ai }\left\{ \left[ \frac{\mathrm{i} \overline{P}^\prime \left( y_0 \right) }{\varepsilon }\right] ^{1/3} \left( y-y_0\right) \right\} + b_0 \text{ Ai }\left\{ \mathrm {e}^{2 \mathrm{i} \pi /3} \left[ \frac{\mathrm{i} \overline{P}^\prime \left( y_0 \right) }{\varepsilon }\right] ^{1/3} \left( y-y_0\right) \right\} , \end{aligned}$$

28c $$\begin{aligned} W_\mathrm{w}= & {} \frac{a_\mathrm{w}}{\left[ -\mathrm{i} \overline{P}\left( y \right) \right] ^{1/4}} \exp \,\left[ \frac{\mathrm{i}^{3/2}}{\sqrt{\varepsilon }} \kappa \left( y \right) \right] + \frac{b_\mathrm{w}}{\left[ -\mathrm{i} \overline{P}\left( y \right) \right] ^{1/4}} \exp \,\left[ -\frac{\mathrm{i}^{3/2}}{\sqrt{\varepsilon }} \kappa \left( y \right) \right] , \end{aligned}$$

and the branch cut is chosen so that

29 $$\begin{aligned} \kappa \left( y \right) = {\left\{ \begin{array}{ll} \int _{y_0}^y \sqrt{\overline{P}\left( \check{y} \right) }\,\,\mathrm {d}\check{y} = \frac{1}{2}\left( y - 1\right) \sqrt{\overline{P}\left( y \right) } + \frac{\sqrt{\pi } \left( 3 - 2 U\right) }{2\sqrt{3}} \left\{ \frac{\pi }{2} - \arcsin \left[ \frac{\sqrt{3} \left( 1 - y\right) }{\sqrt{3 - 2 U}}\right] \right\} &{}\quad \text{ for }~y>y_0, \\ -\int _y^{y_0} \sqrt{-\overline{P}\left( \check{y} \right) }\,\,\mathrm {d}\check{y} = \frac{1}{2} \left( y - 1\right) \sqrt{-\overline{P}\left( y \right) } + \frac{\sqrt{\pi } \left( 3 - 2 U\right) }{2 \sqrt{3}} \ln \left[ \frac{\pi \sqrt{3 - 2 U}}{\sqrt{3} \pi \left( 1 - y\right) - \sqrt{-\pi \overline{P}\left( y \right) }}\right] &{}\quad \text{ for }~y<y_0. \end{array}\right. } \end{aligned}$$

The solution $W_\mathrm{m}(y)$ in (27) occupies $y - y_0 > \varepsilon ^{1/3}$ and is bounded above by the centreline, while $W_\mathrm{w}(y)$ in (27) occupies $y - y_0< - \varepsilon ^{1/3}$ and is bounded below by the channel wall. The solution $W_0(y)$ in (27) is about the turning point at $y = y_0$. $W_{0\mathrm{w}}^\mathrm{c}\left( y \right)$ is the common part matching the behaviour near the turning point to the WKBJ behaviour near the wall, and $W_{0\mathrm{m}}^\mathrm{c}\left( y \right)$ is the common part matching the behaviour near the turning point to the WKBJ behaviour near the centreline. These common parts and $W_0(y)$ are derived in Appendix A. As detailed in Appendices B and C, the constants $a_\mathrm{m}$, $b_\mathrm{m}$, $a_0$, $b_0$, $a_\mathrm{w}$, and $b_\mathrm{w}$ are determined by matching the solutions asymptotically and applying the boundary conditions $W\left( 0 \right) = 1$ and $W^\prime \left( 1 \right) = 0$.

As shown in Appendix C, the asymptotic solution (27) shows excellent agreement with the numerical solution. The changing nature of the flow on either side of the turning point can be explained by the changes in the dominant balances in (11). In this parameter range, the dominant behaviour close to the wall is governed by a balance between the unsteady convection and the wall-normal viscous stresses. Moving away from the wall, the streamwise Poiseuille-driven convection increases. Between the wall and the turning point, the streamwise Poiseuille-driven convection remains smaller than the convection resulting from unsteady wall wave forcing. At the turning point, a constant-viscous-stress balance exists between the streamwise Poiseuille-driven convection and the convection due to the unsteady wave forcing. Above the turning point the convection due to the Poiseuille flow is more significant than the contribution due to the wall wave forcing and the *y* viscous diffusion is present again.

## Langer theory

An asymptotic approximation which is alternative to the WKBJ turning-point solution can be constructed via the Langer transformation: $\eta = \eta (y)$ and $v = \sqrt{\eta ^\prime \left( y \right) } W\left( y \right)$ [44]. This is uniformly valid between the walls and the centreline. Equation (26) can be written as

30 $$\begin{aligned} \frac{\mathrm {d} ^2v}{\mathrm {d} \eta ^2} - \frac{\mathrm{i} \overline{P}}{\varepsilon \left( \eta ^\prime \right) ^2} v = \varDelta v, \quad \text{ where } \quad \varDelta = - \frac{3 \left( \eta ^{\prime \prime }\right) ^2}{4 \left( \eta ^\prime \right) ^4} + \frac{\eta ^{\prime \prime \prime }}{2 \left( \eta ^\prime \right) ^2}. \end{aligned}$$

For $\varepsilon \ll 1$ and $\varDelta = O\,(1)$, the approximate solution to (30) can be calculated from the related equation

$$\begin{aligned} \frac{\mathrm {d} ^2v}{\mathrm {d} \eta ^2} - \frac{\mathrm{i} \overline{P}}{\varepsilon \left( \eta ^\prime \right) ^2} v \sim 0. \end{aligned}$$

**Fig. 10:**
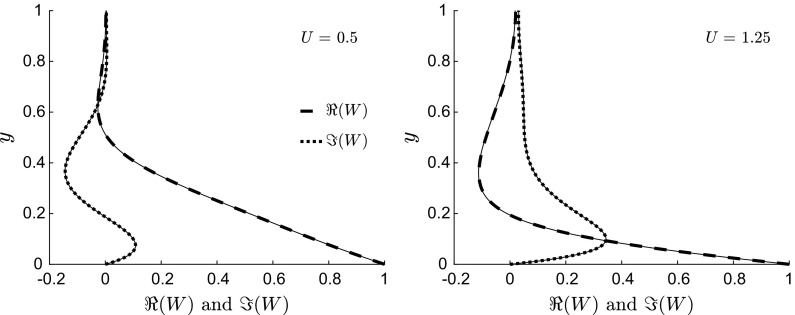
Comparison between the real (thick dashed lines) and imaginary (thick dotted lines) components of the Langer solution (31) with the numerical solutions (thin solid lines) for $\lambda =50$, $R=1000$ and $U=0.5$ (left, as Fig. 6(top left) and case 2 in Sect. 6.1), and $U=1.25$ (right, as Fig. 6 (bottom right) and case 3 in Sect. 6.1). $\varepsilon =0.05$ in both cases

Following Langer [36, 37], we choose $\mathrm{i} \overline{P}/\left( \eta ^\prime \right) ^2 = \eta$, and hence, upon integration,

$$\begin{aligned} \eta (y) = {\left\{ \begin{array}{ll} \left[ \frac{3}{2} \sqrt{\mathrm{i}} \kappa \left( y \right) \right] ^{2/3} &{}\quad \text{ for } \quad y > y_0, \\ -\left[ -\frac{3}{2} \sqrt{\mathrm{i}} \kappa \left( y \right) \right] ^{2/3} &{}\quad \text{ for } \quad y < y_0,\end{array}\right. } \end{aligned}$$

where $\kappa \left( y \right)$ is given by (29). A uniform asymptotic approximation to $W\left( y \right)$ is given by

31 $$\begin{aligned} W\left( y \right) = \left[ -\frac{\mathrm{i} \eta (y)}{\overline{P}\left( y \right) }\right] ^{1/4} \left\{ a_{\text {L}} \text{ Ai }\left[ \varepsilon ^{-1/3} \eta (y)\right] + b_{\text {L}} \text{ Bi }\left[ \varepsilon ^{-1/3} \eta (y)\right] \right\} , \end{aligned}$$

where

32 $$\begin{aligned} \left\{ a_{\text {L}},b_{\text {L}}\right\} = \left[ \frac{\mathrm{i} \overline{P}\left( 0 \right) }{\eta \left( 0 \right) }\right] ^{1/4} \frac{\left\{ \text{ Bi }^\prime \left( \eta _1\right) ,-\text{ Ai }\left( \eta _1 \right) \right\} }{\text{ Ai }{\,}\left( \eta _0 \right) \text{ Bi }^\prime \left( \eta _1 \right) - \text{ Ai }^\prime \left( \eta _1 \right) \text{ Bi }\,\left( \eta _0 \right) } \end{aligned}$$

and $\eta _0=\varepsilon ^{-1/3} \eta \left( 0 \right)$ and $\eta _1=\varepsilon ^{-1/3} \eta \left( 1 \right)$. The real and imaginary parts of the Langer solution (31), shown in Fig. 10 for two cases investigated in Sect. 6.1, show excellent agreement with the numerical profiles.

## Application to flow mixing

It is our hope that our theoretical results will spur experimental research for which the fluid-structure interaction driven by shear-horizontal waves is important. In this section, we put forward ideas for their possible future applications.

**Fig. 11:**
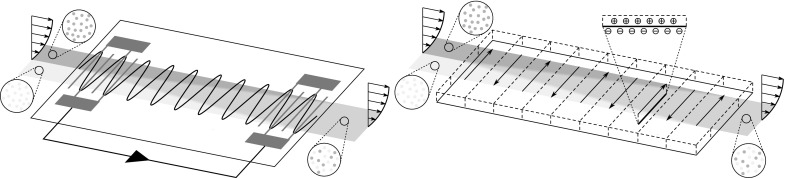
Schematic drawings of mixers of two microfluidic flows at different passive scalar concentrations. Left: forcing by a shear-horizontal surface acoustic wave. Right: forcing by an electro-osmotic wall wave

**Fig. 12:**
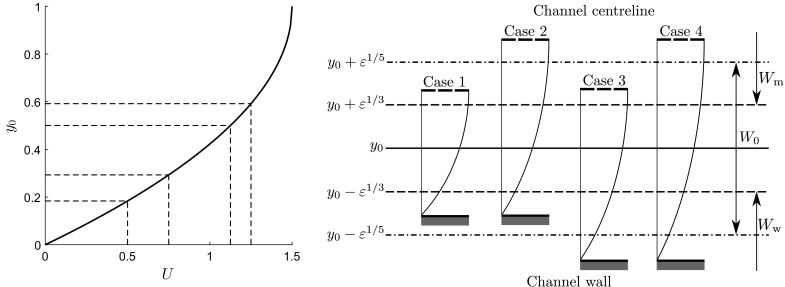
Left: Turning-point position $y_0$ as a function of *U*. The dotted lines represent the flows shown in Fig. 6. Right: Flow configurations for the turning-point solution composite expansion, the channel wall (shaded region) and the channel centreline (thick dashed line)

- *Active mixing of laminar flows by surface acoustic waves.*
  Rayleigh waves, i.e. wall-normal displacement streamwise-travelling acoustic waves, have been utilized for microfluidic mixing. Sritharan et al. [1] and Tseng et al. [45] experimentally verified that Rayleigh waves can mix cofluent streams with very different passive scalar concentrations. However, in liquids these waves suffer from severe energy dissipation due to the compression waves engendered by the wall-normal displacement (leaky-wave phenomenon). Also, although the induced small-scale secondary recirculatory motion is beneficial for mixing, it may interfere with the smoothness of the streamwise flow and create additional pressure gradients and therefore additional losses. We instead propose to use shear-horizontal waves to mix the cofluent streams studied by Sritharan et al. [1] and Tseng et al. [45], which, to the best of our knowledge, have never been employed in microfluidic mixing. A schematic of the microfluidic SAW mixer is shown in Fig. 11(left).
  The main advantages over the Rayleigh waves would be (i) less energy dissipation (higher efficiency) because the shear-horizontal waves do not suffer from acoustic streaming energy loss due to the absence of wall-normal motion, as thoroughly discussed by Lange et al. [13] and Rocha et al. [46], which implies (ii) mixing along longer streamwise distances [46]. Furthermore, (iii) as the mixing occurs through the spanwise velocity, the streamwise flow remains smooth and no additional induced pressure gradients must be accounted for. A fourth advantage is that (iv) the spanwise waves would be better mixers than two-dimensional Rayleigh waves given the concentration distribution at the inlet, shown in Fig. 11(left), which is uniform along the wall-normal direction, but strongly varying along the spanwise direction. Although the streamlines of the streamwise flow are unchanged when the SAWs are implemented, the mixing is required primarily along the spanwise direction where the concentration variations are most intense.
  The passive scalar equation to be solved is
  33 $$\begin{aligned} \frac{\partial \theta }{\partial t} + u \frac{\partial \theta }{\partial x} + \boxed {w \frac{\partial \theta }{\partial z}} = \frac{1}{\mathrm{Pe}} \left( \frac{\partial ^2 \theta }{\partial x^2} + \frac{\partial ^2 \theta }{\partial y^2} + \frac{\partial ^2 \theta }{\partial z^2}\right) , \end{aligned}$$
  where $\theta$ is the passive scalar concentration (mass or temperature, for example), $\mathrm{Pe}=U_\mathrm{b}^* h^*/\alpha _\mathrm{p}^*$ is the Peclet number, and $\alpha _\mathrm{p}^*$ is the diffusion coefficient for mass transfer and the thermal diffusivity for heat transfer. The spanwise waves *w*(*x*, *y*, *z*, *t*) would act, through the boxed term in (33), on the spanwise gradient of $\theta$, which would be most intense at the upstream channel location, where the two cofluent streams start interacting. The Reynolds numbers in the experiments of Sritharan et al. [1] and Tseng et al. [45] are in the range $10^{-2}-1$ and the forcing wavelengths are comparable or smaller than the channel height, which leads to $\varepsilon =\lambda /R$ in the range 1–100. This corresponds to the order-one-$\varepsilon$ and large-$\varepsilon$ regimes with *U* belonging to any columns of Fig. 8 because a wide range of wave speeds can be generated by wave interference, as proved by the standing- wave study by Ding et al. [8].
  We finally note that the boundary-layer analysis of Sect. 5 is also here useful because it allows identifying the regimes where the spanwise flow is confined very near the wall, which are bound not to be efficient mixers as the mixing is required across the whole channel height. The turning-point regimes, for which the wave speed is comparable with the bulk velocity, could instead qualify as candidates for good mixing because the flow may extend along the channel height (refer to Fig. 6).
- *Active mixing of laminar flows by electro-osmotic waves.*
  The travelling wave flow produced by (1) could also be generated by electro-osmotic waves and used for microfluidic flow mixing, as shown in Fig. 11(right). To the best of our knowledge, these waves have never been created through electro-osmosis, but proper design of an unsteady and spatially inhomogeneous electric field could achieve this purpose. They would be an unsteady and streamwise-modulated version of the waves suggested by Ajdari [18], a streamwise-modulated variant of the oscillatory flow studied numerically by Dutta and Beskok [47], or an optimized variant of the micromixer proposed by Oddy et al. [23], where the wall forcing would be spanwise and streamwise-modulated instead of simply oscillatory and spatially uniform. Extending the analyses of Dutta and Beskok [47] and Meisel and Ehrhard [25], the governing equation for the spanwise velocity with electro-osmotic effects included is
  34 $$\begin{aligned} \frac{\partial w^*}{\partial t^*} + u^* \frac{\partial w^*}{\partial x^*} = \nu ^* \left( \frac{\partial ^2 w^*}{\partial x^{*2}} + \frac{\partial ^2 w^*}{\partial y^{*2}} \right) - \frac{\overline{\varepsilon }^* \zeta ^* E_\mathrm{z}^*(x^*,t^*)}{\ell _\mathrm{d}^{*2}\rho ^*} \mathrm {e}^{-y^*/\ell _\mathrm{d}^*}, \end{aligned}$$
  subject to the no-slip boundary condition at the wall and to the zero-gradient condition at the centreline (we only consider half channel for simplicity). Here $\ell _\mathrm{d}^*$ is the thickness of the Debye–Hückel layer, $E_\mathrm{z}^*=E_{\mathrm{z},0}^*\mathfrak {R} \big [\mathrm {e}^{2 \pi \mathrm{i} \left( x^* - U^* t^* \right) /\lambda ^*} \big ]$ is the spanwise electric field, $\overline{\varepsilon }^*$ is the permittivity, and $\zeta ^*$ is the zeta potential (we have set the ionic energy parameter equal to unity, refer to Dutta and Beskok [47, p. 5098], and for simplicity assumed an exponentially decaying potential as in Meisel and Ehrhard [25] rather than more complex potential functions as in Afonso et al. [48] and Wang et al. [49]). The details of the formulation for the Stokes layer case, i.e. for $u^*=0$ and $E_\mathrm{z}^*=E_{\mathrm{z},0}^*\mathfrak {R} \big ( \mathrm {e}^{\mathrm{i} \omega ^* t^*} \big )$, are found in Dutta and Beskok [47].
  The above problem can be conveniently simplified under the assumption that Debye–Hückel layer $\ell _\mathrm{d}^*$ is much thinner than the channel height and the viscous layers studied in Sect. 5. As also discussed by Qiao and Aluru [50], this hypothesis is amply verified as $\ell _\mathrm{d}^*$ is very small, i.e. of the order of  $100~\hbox {nm}$ [47, 51], therefore about three orders of magnitude smaller than the channel height and two orders smaller than the viscous layers generated by the travelling waves. This means that the electric potential is confined in this very thin near-wall Debye–Hückel layer, while the bulk flow is electrically neutral and driven underneath by the electro-osmotic motion of the Debye–Hückel layer. The scaled form of (34) is
  35 $$\begin{aligned} \frac{\partial w}{\partial t} + \frac{u}{U} \frac{\partial w}{\partial x} = \frac{1}{\lambda R U} \frac{\partial ^2 w}{\partial x^2} + \frac{\lambda }{R U} \frac{\partial ^2 w}{\partial y^2} - \frac{\varPi _\mathrm{z}}{R \delta _\mathrm{d}^2} \mathrm {e}^{-y/\delta _\mathrm{d}}, \end{aligned}$$
  where $\varPi _\mathrm{z}=\overline{\varepsilon }^* \zeta ^* E_\mathrm{z}^* \lambda ^*/(\mu ^* h^* U^*)=O(1)$, $\mu ^*$ is the dynamic viscosity of the fluid, $\delta _\mathrm{d}=\ell _\mathrm{d}^*/h^*\ll 1$, and $\lambda ,U,R=O(1)$. Note that here $w=w^*/U_\mathrm{b}^*$ as $A^*$ cannot be used for scaling like in Sect. 2 because it is not defined. It is found in the following through asymptotic matching. By defining the Debye–Hückel-layer coordinate $Y_\mathrm{d}=y/\delta _\mathrm{d}$ and velocity $W_\mathrm{d}=w$, the Debye–Hückel-layer equation is found at leading order
  36 $$\begin{aligned} \frac{\partial ^2 W_\mathrm{d}}{\partial Y_\mathrm{d}^2}=\frac{U \varPi _\mathrm{z}}{\lambda } \mathrm {e}^{-Y_\mathrm{d}}, \end{aligned}$$
  whose solution is
  37 $$\begin{aligned} W_\mathrm{d}(x,Y_\mathrm{d},t)=\frac{U \varPi _\mathrm{z}}{\lambda }\Big ( \mathrm {e}^{-Y_\mathrm{d}} - 1 \Big ), \end{aligned}$$
  obtained by use of the boundary conditions $W_\mathrm{d}(0)=0$ and $W_\mathrm{d}'(\infty )=0$. The Helmholtz–Smoluchowski velocity is obtained as follows:
  38 $$\begin{aligned} W_{\mathrm{hs}}(x,t) = \lim _{Y_\mathrm{d} \rightarrow \infty }W_\mathrm{d}(x,Y_\mathrm{d},t)=-\frac{U \varPi _\mathrm{z}}{\lambda }. \end{aligned}$$
  In dimensional form, (38) becomes $W_{\mathrm{hs}}^*=-\big (\overline{\varepsilon }^* \zeta ^*E_{\mathrm{z},0}^*/\mu ^*\big )\mathfrak {R} \big [\mathrm {e}^{2 \pi \mathrm{i} \left( x^* - U^* t^* \right) /\lambda ^*} \big ]$. This is the velocity that drives the bulk electrically neutral flow which we have studied in the previous sections. We can now quantify the amplitude of the wall travelling waves defined in (3), i.e. $A^*=-\overline{\varepsilon }^* \zeta ^*E_{\mathrm{z},0}^*/\mu ^*$. In summary, the bulk spanwise flow, which is relevant for mixing, is thus described by Eq. (4) and driven by the unsteady and streamwise-modulated Helmholtz–Smoluchowski velocity $W_{\mathrm{hs}}$ in (38), while the spanwise velocity in the very thin Debye–Hückel layer is brought to zero at the wall through the no-slip condition. The composite solution is $w_\mathrm{c}(x,y,t)=w(x,y,t)+W_\mathrm{d}(x,Y_\mathrm{d},t)-W_{\mathrm{hs}}(x,t)$.
  As for the SAW mixer, the passive scalar Eq. (33) is to be solved. Similar electro-osmotic microfluidic mixers have been studied by Sasaki et al. [52] and Huang et al. [53]. Sasaki et al. [52] employed meandering electrodes to mix two microstreams and stress the importance of obtaining analytical results for the fluid flow in order to optimize the mixing performance, while Huang et al. [53] generated in-plane microvortices to prove that up to 30-fold mixing enhancement can be achieved compared to mixing due to diffusion only. The spanwise spatial pattern displayed in Fig. 11(right) can be achieved by utilizing thin strips of different glass coatings and spatially modulated electric fields [18, 20].
  Typical frequencies of electro-osmosis actuators are of the order of 10 Hz [23, 53] and the streamwise length of the microelectrode arrays, which define the forcing wavelength, can be in the range of 100–$500~\upmu \hbox {m}$. The flow parameters therefore correspond to ratios $\varepsilon =\lambda /R$ in the range of 0.1–1 and to *U* of order unity. The small-$\varepsilon$ and order-one-$\varepsilon$ regimes characterize these flows. The turning-point WKBJ analysis and the Langer analysis are thus relevant for these flow regimes, where the wave speeds are positive and comparable with the bulk velocity. The streamwise wall-shear stress would play a crucial role in the spanwise flow dynamics and thus for the mixing performance. The design proposed in Fig. 11(right) would generate a square streamwise-travelling wave of spanwise velocity, a micro-scale analogous version of the pipe-flow wave employed by Auteri et al. [28]. As explained in Sect. 9, thanks to the linearity of the problem, Fourier decomposition will allow the use of our sinusoidal-wave results to construct the base flow for this mixing problem.

As mentioned in the Introduction, the shear-horizontal waves have also been used for turbulent drag reduction. For this application, the phase speeds span a wide range of values from null to several times the bulk streamwise velocity. It therefore follows that the regime with $\varepsilon =\lambda /R \ll 1$ properly describes these flows. It also occurs that $\lambda \gg R^{-1/2}$ and therefore the streamwise viscous diffusion due to the waves is negligible. We reiterate that, in fully developed turbulent channel flows, the laminar flow solutions may only be representative of the spanwise-averaged velocity profile and only under strict conditions of the wave parameters, as amply discussed in Quadrio and Ricco [32]. It is interesting to note that the drag-increase regime discovered by Quadrio et al. [31] for a specific range of positive phase speeds is included within the range for which the turning-point regime occurs. Further investigation is also required to generalize the stability analyses for the classical Stokes layer [54, 55] to the travelling wave case.

## Summary

Channel flows with spanwise wall forcing consisting of in-phase sinusoidal travelling waves of spanwise wall velocity have been investigated. A novel three-dimensional time-dependent solution of the incompressible Navier–Stokes equations is constructed, with the solution represented as a linear combination of complex-to-complex parabolic cylinder functions. As reliable numerical solutions of the complex-to-complex parabolic cylinder function are currently unavailable, asymptotic solution methods have been employed to investigate flow variations due to the Reynolds number *R*, the scaled phase speed *U*, and the scaled wavelength $\lambda$. Only sinusoidal shear-horizontal wall forcing has been considered. However, flows produced by more general wall forcing can be expressed by a linear combination of our solutions with each term multiplied by its Fourier coefficient.

Asymptotic methods, i.e. the boundary-layer and the WKBJ theories, have been utilized to study the flow. The underlying flow physics, revealed by the dominant balances in the governing equation, is gained by using these asymptotic theories and cannot be obtained through the exact analytical solution (10) or the numerical solutions computed in Sect. 4. While the boundary-layer method and the WKBJ approach both produce excellent approximations to the flow, there are particular advantages for each method. The simplicity of the boundary-layer solutions compared to the WKBJ solution is noticeable, aided by the outer solutions being identically zero. The boundary-layer method readily enables the determination of the boundary-layer thickness, which is not available if the WKBJ method is employed. Furthermore, the link between the different physical effects is elucidated better utilizing the boundary-layer approach. There are also advantages of the WKBJ approach over the boundary-layer method. Without further scaling (introducing a second boundary layer close to $y=2$), the WKBJ method readily generates solutions which are valid across the full channel.

The WKBJ solution also identifies the turning-point behaviour for $0 \le U \le 3/2$, $R^{-1/2} \ll \lambda \ll R$, which is not explained by the boundary-layer method. As the standard WKBJ solution (25) is unbounded near the turning point, solutions have been found by a WKBJ composite expansion and the Langer method. While the Langer solution is simpler, the composite WKBJ expansion has the benefits of explicitly determining the turning-point location and of quantifying the thickness of this thin layer. This is important because the physics changes there. The WKBJ theory shows that, when the streamwise diffusion effects are negligible, in the turning-point layer the Poiseuille convection and the convection due to the waves cancel out so that the wall-normal viscous stresses are constant. The high accuracy of the asymptotic solutions is quantified by comparing them with the numerical profiles in Appendix D.

We have finally presented ideas on how to generate the travelling waves for microfluidic flow mixing via surface acoustic forcing and electro-osmotic actuation. In the latter case, matched asymptotic expansion has been useful to obtain the Helmholtz–Smoluchowski velocity that drives the bulk spanwise flow.

## Figures and Tables

**Fig. 13:**
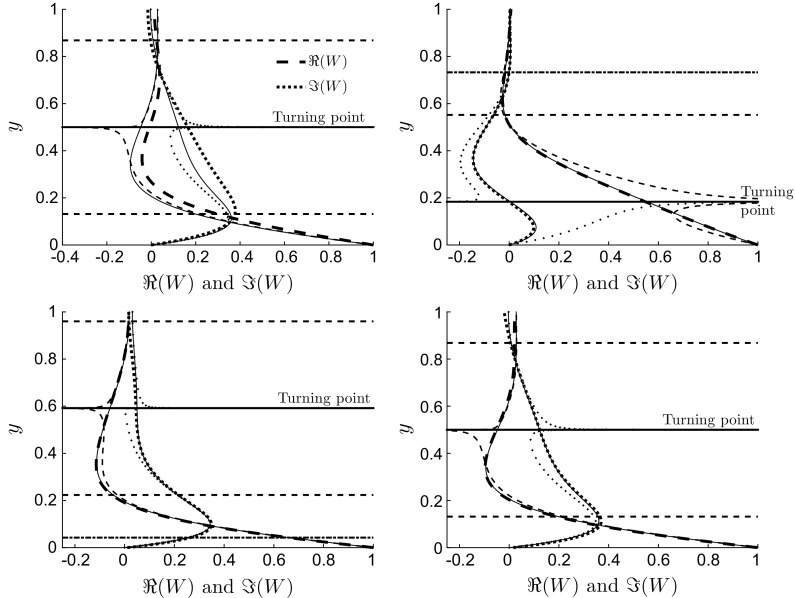
Profiles of $W\left( y \right)$ for $\lambda =50$, $R=1000$ and case 1 with $U=1.125$, (top left), case 2 with $U=0.5$ (top right), case 3 with $U=1.25$ (bottom left), and case 4 with $U=1.125$. The real (thick dashed) and imaginary (thick dotted) components of the composite expansion are shown, along with the breakdown of the real (thin dashed), and imaginary (thin dotted) components of WKBJ solution (23) close to the turning point, and the numerical solutions (solid curves). The horizontal solid lines indicate the position of the turning points $y_0$, while the horizontal dashed lines (at $y_0\pm \left( \lambda /R\right) ^{1/3}$) and the horizontal dash-dotted lines (at $y_0\pm \left( \lambda /R\right) ^{1/5}$) mark the boundaries of the matching regions

**Fig. 14:**
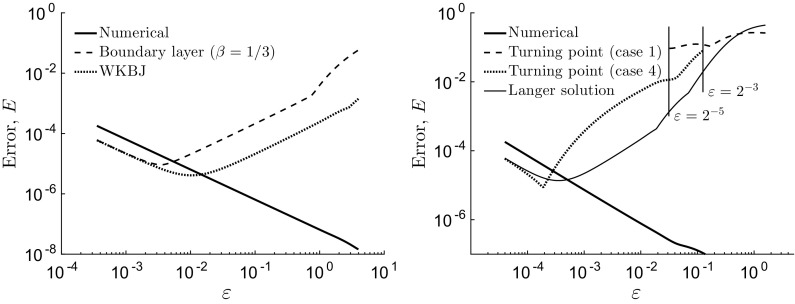
Left: The error *E* calculated using the boundary-layer method (dashed line), and the WKBJ approximation (dotted line) for $U=10$. Right: The error *E* calculating using the turning-point method (case 1, dashed line, case 4, dotted line), and the Langer method (thin solid line) for $U=1.125$. In both figures, the error in numerical solution on two different meshes is shown by the thick solid line

## Appendix A: Common parts in WKBJ turning-point composite expansion

The common part $W_{0\mathrm{m}}^\mathrm{c}\left( y \right)$, of the matched asymptotic expansion of $W_0\left( y \right)$ and $W_\mathrm{m}\left( y \right)$, which is valid in $\varepsilon ^{1/3}< y - y_0 < \varepsilon ^{1/5}$, is given by

39a $$\begin{aligned} W_{0\mathrm{m}}^\mathrm{c} = \left[ \mathrm{i} \overline{P}^\prime \left( y_0 \right) (y - y_0)\right] ^{-1/4} \left\{ a_\mathrm{m} \exp \left[ -\frac{2}{3} \sqrt{\frac{\mathrm{i} \overline{P}^\prime \left( y_0 \right) }{\varepsilon }} \left( y - y_0\right) ^{3/2}\right] + b_\mathrm{m} \exp \left[ \frac{2}{3} \sqrt{\frac{\mathrm{i} \overline{P}^\prime \left( y_0 \right) }{\varepsilon }} \left( y - y_0\right) ^{3/2}\right] \right\} , \end{aligned}$$

while the common part $W_{0\mathrm{w}}^\mathrm{c}\left( y \right)$ of the matched asymptotic expansion of $W_0\left( y \right)$ and $W_\mathrm{w}\left( y \right)$, which is valid in $-\varepsilon ^{1/5}< y-y_0 < -\varepsilon ^{1/3}$, is given by

39b $$\begin{aligned} W_{0\mathrm{w}}^\mathrm{c} = \frac{\mathrm {e}^{3 \mathrm{i}\pi /4}}{\left[ -\mathrm{i} \overline{P}^\prime \left( y_0 \right) \left( y - y_0\right) \right] ^{1/4}} \left\{ a_\mathrm{w} \exp \left[ -\frac{2}{3} \sqrt{-\frac{\mathrm{i} \overline{P}^\prime \left( y_0 \right) }{\varepsilon }} \left( y -y_0\right) ^{3/2}\right] + b_\mathrm{w} \exp \left[ \frac{2}{3}\sqrt{-\frac{\mathrm{i} \overline{P}^\prime \left( y_0 \right) }{\varepsilon }} \left( y - y_0\right) ^{3/2}\right] \right\} .\nonumber \\ \end{aligned}$$

Here the branch cuts are chosen so that

$$\begin{aligned} \left( y - y_0\right) ^{1/4} = {\left\{ \begin{array}{ll} \left( y - y_0\right) ^{1/4}, &{}\quad y> y_0, \\ -\left( y_0 - y\right) ^{1/4}, &{} \quad y< y_0, \end{array}\right. } \quad \text{ and } \quad \left( y - y_0\right) ^{3/2} = {\left\{ \begin{array}{ll} \left( y - y_0\right) ^{3/2}, &{}\quad y > y_0, \\ -\left( y_0 - y\right) ^{3/2}, &{}\quad y < y_0. \end{array}\right. } \end{aligned}$$

The solution around the turning point, $W_0\left( y \right)$, is constructed by employing a new transition layer coordinate $\hat{y}= \varepsilon ^{-1/3} \left( y - y_0\right)$ and by expanding $\overline{P}\left( y \right)$ in (26) about $y=y_0$ to give

40 $$\begin{aligned} \overline{P}\left( y \right) = \overline{P}\left( y_0 \right) + \varepsilon ^{1/3} \hat{y}\overline{P}^\prime \left( y_0 \right) + \frac{\varepsilon ^{2/3} \hat{y}^2}{2} \overline{P}^{\prime \prime }\left( y_0 \right) + \cdots . \end{aligned}$$

As $\overline{P}\left( y_0 \right) = 0$ and $\overline{P}^\prime \left( y_0 \right) > 0$ for $y_0 \in \left[ 0,\,1\right)$, in cases where the linear term in (40) is much larger than the quadratic term, the leading-order behaviour of Eq. (26) satisfies the Airy equation

$$\begin{aligned} \frac{\mathrm {d} ^2 W_0}{\mathrm {d} \hat{y}^2} - \mathrm{i} \overline{P}^\prime \left( y_0 \right) \hat{y}W_0\left( \hat{y} \right) =0, \end{aligned}$$

and hence the solution (28b) is obtained around the turning point. The analysis is not complete until the three expressions (28a)–(28c) are asymptotically matched in the regions where they overlap. As described in Appendix B, linearly independent solutions of the Airy equation, $\text{ Ai }\,\left( y \right)$ and $\text{ Ai }\,\left( \mathrm {e}^{2\mathrm{i}\pi /3} y \right)$, are utilized to facilitate the matching with the outer WKBJ behaviour above and below the turning-point region.

The turning-point behaviour given by (28b) is not valid when the quadratic term in (40) is as large as the linear term and hence cannot be neglected. This occurs when $y_0$ is close to the centreline because $\overline{P}^\prime \left( 1 \right) = 0$. As $\hat{y}=O(1)$, the linear and the quadratic terms balance when $y_0-1=O\,(\varepsilon ^{1/3})$, which is found by use of the definition of $\overline{P}\left( y \right)$. Inside this region, the quadratic term in the expansion (40) cannot be neglected and, rather than the Airy equation, the local behaviour is governed by an ordinary differential equation whose solutions are parabolic cylinder functions. Consistent with our earlier approach of not developing an algorithm for complex-to-complex parabolic cylinder functions, the particular case with the turning point close to the centreline will not be discussed further.

## Appendix B: Matching for WKBJ turning-point composite expansion

When the solution about the turning point is given by (28b), $W_0$ and $W_\mathrm{m}$ are matched over a region $\varepsilon ^{1/3}< y-y_0 < \varepsilon ^{1/5}$, which gives rise to the connection formulae:

41 $$\begin{aligned} a_\mathrm{m} = \frac{a_0 \varepsilon ^{1/12} \left[ \mathrm{i} \overline{P}^\prime \left( y_0 \right) \right] ^{1/6}}{2 \sqrt{\pi }} \quad \text{ and } \quad b_\mathrm{m} = \frac{b_0 \varepsilon ^{1/12} \left[ \mathrm{i} \overline{P}^\prime \left( y_0 \right) \right] ^{1/6} \mathrm {e}^{\mathrm{i}\pi /6}}{2 \sqrt{\pi }}. \end{aligned}$$

Similarly, $W_\mathrm{w}$ and $W_0$ are matched over a region $-\varepsilon ^{1/5}< y-y_0 < -\varepsilon ^{1/3}$, which leads to

42 $$\begin{aligned} a_\mathrm{w} = \frac{a_0 \varepsilon ^{1/12} \left[ \mathrm{i} \overline{P}^\prime \left( y_0 \right) \right] ^{1/6} \mathrm {e}^{\mathrm{i} \pi /4}}{2 \sqrt{\pi }} \quad \text{ and } \quad b_\mathrm{w} = \frac{b_0 \varepsilon ^{1/12} \left[ \mathrm{i} \overline{P}^\prime \left( y_0 \right) \right] ^{1/6} \mathrm {e}^{\mathrm{i} \pi /12}}{2 \sqrt{\pi }}. \end{aligned}$$

The derivation of the connection formulae is usually given for cases where $\mathrm{i} \overline{P}\left( y \right)$ is a strictly real valued function and is found in Bender and Orszag [38]. The derivation is extended now to the problem of a strictly complex-value $\mathrm{i} \overline{P}\left( y \right)$. For $y>y_0$, the Taylor expansion of $\overline{P}\left( y \right)$ about $y=y_0$ is used in the integral for $\kappa \left( y \right)$, and hence

$$\begin{aligned} \kappa \left( y \right) = \int _{y_0}^y \sqrt{\left( \tilde{y}-y_0\right) \overline{P}^\prime \left( y_0 \right) + \frac{1}{2} \left( \tilde{y}-y_0\right) ^2 \overline{P}^{\prime \prime }\left( y_0 \right) } \,\,\mathrm {d}\tilde{y}. \end{aligned}$$

As previously discussed, when $y=y_0$ is sufficiently far from $y=1$, the quadratic term can be neglected in favour of the linear term and hence $\kappa \left( y \right) \approx (2/3) \sqrt{\varepsilon } r^{3/2}$, where $r = \varepsilon ^{- 1/3} \left( \mathrm{i} P_0^\prime \right) ^{1/3} \left( y - y_0\right)$ is the variable in the matching region $\varepsilon ^{1/3}< y-y_0 < \varepsilon ^{1/5}$. Similarly, in this regime $\left[ \mathrm{i} P\left( y \right) \right] ^{-1/4} = \varepsilon ^{-1/12} \left( \mathrm{i} P_0^\prime \right) ^{-1/6} r^{-1/4}$, and hence for $y - y_0 \rightarrow 0+$, the WKBJ solution (28c) can be written as

43 $$\begin{aligned} W_\mathrm{m} \sim \frac{\varepsilon ^{-1/12}}{\left( \mathrm{i} P_0^\prime \right) ^{1/6} r^{1/4}} \left[ a_\mathrm{m} \exp \,\left( -\frac{2}{3} r^{3/2} \right) + b_\mathrm{m} \exp \,\left( \frac{2}{3} r^{3/2} \right) \right] . \end{aligned}$$

This solution needs to be matched with the large *y* behaviour of the local turning-point solution (28b). The asymptotic approximation to the Airy function is [38]

44 $$\begin{aligned} \text{ Ai }\left( z \right) \sim \frac{1}{2 \sqrt{\pi } z^{1/4}} \exp \,\left( -\frac{2}{3} z^{3/2} \right) \quad \text{ for } \quad \left| z \right| \rightarrow \infty , \quad \text{ and } \quad \left| \arg {z} \right| < \pi . \end{aligned}$$

Consequently for $y \rightarrow \infty$, the local solution about the turning point has the form

45 $$\begin{aligned} W_0 \sim \frac{a_0}{2 \sqrt{\pi } r^{1/4}} \exp \,\left( -\frac{2}{3} r^{3/2} \right) + \frac{b_0}{2 \sqrt{\pi } \mathrm {e}^{\mathrm{i} \pi /6} r^{1/4}} \exp \,\left( \frac{2}{3} r^{3/2} \right) . \end{aligned}$$

Comparing the coefficients of the exponential terms in (43) and (45) gives rise to the connection formula (41), while similar matching between (28c) and (28b) gives rise to the connection formula (42).

## Appendix C: WKBJ turning-point composite solutions

In this Appendix, the possible flow configurations that can arise due to the relationship between the matching regions, the wall and the centreline, are discussed. As illustrated from left to right in Fig. 12(right), one finds:

- *Case 1* ($y_0 < \varepsilon ^{1/5}$ and $y_0 > 1 - \varepsilon ^{1/5}$): $W_0$ is valid across the full half channel, with $W\left( y \right) =W_0\left( y \right)$,
  $$\begin{aligned} a_0 = \frac{\mathrm {e}^{2 \pi \mathrm{i}/3} B_1}{\mathrm {e}^{2 \pi \mathrm{i}/3} A_0 B_1 - A_1 B_0} \qquad \text{ and } \qquad b_0 = -\frac{A_1}{\mathrm {e}^{2 \pi \mathrm{i}/3} A_0 B_1 - A_1 B_0}, \end{aligned}$$
  where $A_0$, $B_0$, $A_1$, and $B_1$ are defined in (46).
- *Case 2* ($y_0 < \varepsilon ^{1/5}$ and $y_0 < 1 - \varepsilon ^{1/3}$): $W_0$ is valid near the wall and the turning point, while $W_\mathrm{m}$ is valid near the centreline. Here $W\left( y \right) = W_\mathrm{m}\left( y \right) + W_0\left( y \right) - W_{0\mathrm{m}}^\mathrm{c}\left( y \right)$, with
  $$\begin{aligned} a_0 = \left\{ A_0 + \exp \left[ -2 \sqrt{\frac{\mathrm{i}}{\varepsilon }} \kappa \left( 1 \right) \right] B_0\right\} ^{-1} \qquad \text{ and } \qquad b_0 = \mathrm {e}^{-\mathrm{i}\pi /6} \left\{ \exp \left[ 2 \sqrt{\frac{\mathrm{i}}{\varepsilon }} \kappa \left( 1 \right) \right] A_0 + B_0\right\} ^{-1}, \end{aligned}$$
  where $A_0$ and $B_0$ are defined in (46).
- *Case 3* ($y_0 > \varepsilon ^{1/3}$ and $y_0 > 1- \varepsilon ^{1/5}$): $W_0$ is valid near the turning point and the centreline, while $W_\mathrm{w}$ is valid near the wall. Here $W\left( y \right) = W_\mathrm{w}\left( y \right) + W_0\left( y \right) - W_{0\mathrm{w}}^\mathrm{c}\left( y \right)$, with
  $$\begin{aligned} a_0 = \frac{2 \sqrt{\pi } \mathrm {e}^{-\mathrm{i}\pi /4} B_1 \left[ -\mathrm{i} \overline{P}\left( 0 \right) \right] ^{1/4}}{\varepsilon ^{1/12} \left[ \mathrm{i} \overline{P}^\prime \left( y_0 \right) \right] ^{1/6} \left\{ B_1 \exp \left[ \frac{\mathrm{i}^{3/2}}{\sqrt{\varepsilon }} \kappa \left( 0 \right) \right] + A_1 \exp \left[ \frac{\mathrm{i} \pi }{6} - \frac{\mathrm{i}^{3/2}}{\sqrt{\varepsilon }} \kappa \left( 0 \right) \right] \right\} } \end{aligned}$$
  and
  $$\begin{aligned} b_0 = \frac{2 \sqrt{\pi } \mathrm {e}^{\mathrm{i}\pi /12} A_1 \left[ -\mathrm{i} \overline{P}\left( 0 \right) \right] ^{1/4}}{\varepsilon ^{1/12} \left[ \mathrm{i} \overline{P}^\prime \left( y_0 \right) \right] ^{1/6}\left\{ B_1 \exp \left[ \frac{\mathrm{i}^{3/2}}{\sqrt{\varepsilon }} \kappa \left( 0 \right) \right] + A_1 \exp \left[ \frac{\mathrm{i} \pi }{6} - \frac{\mathrm{i}^{3/2}}{\sqrt{\varepsilon }} \kappa \left( 0 \right) \right] \right\} }, \end{aligned}$$
  where $A_1$ and $B_1$ are defined in (46).
- *Case 4* ($y_0 > \varepsilon ^{1/3}$ and $y_0 < 1 - \varepsilon ^{1/3}$): $W_0$ is valid near the turning point, while $W_\mathrm{w}$ is valid near the wall and $W_\mathrm{m}$ is valid near the centreline. Here $W\left( y \right)$ is given by (27), with
  $$\begin{aligned} a_0 = \frac{2\sqrt{\pi } \exp \left[ 2 \sqrt{\frac{\mathrm{i}}{\varepsilon }} \kappa \left( 1 \right) \right] \left[ -\mathrm{i} \overline{P}\left( 0 \right) \right] ^{1/4}}{\varepsilon ^{1/12} \left[ \mathrm{i} \overline{P}^\prime \left( y_0 \right) \right] ^{1/6} \left\{ \exp \left[ 2 \sqrt{\frac{\mathrm{i}}{\varepsilon }} \kappa \left( 1 \right) + \frac{\mathrm{i}^{3/2}}{\sqrt{\varepsilon }} \kappa \left( 0 \right) + \frac{\mathrm{i} \pi }{4}\right] + \exp \left[ \frac{\mathrm{i} \pi }{4} - \frac{\mathrm{i}^{3/2}}{\sqrt{\varepsilon }} \kappa \left( 0 \right) \right] \right\} } \end{aligned}$$
  and
  $$\begin{aligned} b_0 = \frac{2\sqrt{\pi } \mathrm {e}^{-\mathrm{i}\pi /6} \left[ -\mathrm{i} \overline{P}\left( 0 \right) \right] ^{1/4}}{\varepsilon ^{1/12} \left[ \mathrm{i} \overline{P}^\prime \left( y_0 \right) \right] ^{1/6} \left\{ \exp \left[ 2 \sqrt{\frac{\mathrm{i}}{\varepsilon }} \kappa \left( 1 \right) + \frac{\mathrm{i}^{3/2}}{\sqrt{\varepsilon }} \kappa \left( 0 \right) + \frac{\mathrm{i} \pi }{4}\right] + \exp \left[ \frac{\mathrm{i} \pi }{4} - \frac{\mathrm{i}^{3/2}}{\sqrt{\varepsilon }} \kappa \left( 0 \right) \right] \right\} }. \end{aligned}$$

Herein

46a $$\begin{aligned} A_0= & {} \text{ Ai }\left\{ -\varepsilon ^{-1/3} \left[ \mathrm{i} \overline{P}^\prime \left( y_0 \right) \right] ^{1/3} y_0\right\} , \quad B_0 = \text{ Ai }\left\{ -\varepsilon ^{-1/3} \mathrm {e}^{2 \pi \mathrm{i}/3} \left[ \mathrm{i} \overline{P}^\prime \left( y_0 \right) \right] ^{1/3} y_0\right\} , \end{aligned}$$

46b $$\begin{aligned} A_1= & {} \text{ Ai }^\prime \left\{ \varepsilon ^{-1/3} \left[ \mathrm{i} \overline{P}^\prime \left( y_0 \right) \right] ^{1/3} \left( 1-y_0\right) \right\} , \quad B_1 = \text{ Ai }^\prime \left\{ \varepsilon ^{-1/3} \mathrm {e}^{2 \pi \mathrm{i}/3} \left[ \mathrm{i} \overline{P}^\prime \left( y_0 \right) \right] ^{1/3} \left( 1-y_0\right) \right\} . \end{aligned}$$

When $y_0 < \varepsilon ^{1/5}$ and $y_0 > \varepsilon ^{1/3}$ the wall is contained within the matching region between $W_\mathrm{w}\left( y \right)$ and $W_0\left( y \right)$, and when $y_0 < 1 - \varepsilon ^{1/3}$ and $y_0 > 1 - \varepsilon ^{1/5}$ the centreline is contained in a matching region between $W_\mathrm{m}\left( y \right)$ and $W_0\left( y \right)$. In these parameter regimes, two different configurations are possible because both a WKBJ solution and the local turning-point solution are valid near one of the boundaries.

Examples of each of the four cases with $\lambda =50$ and $R=1000$ are shown in Fig. 13 with case 1 ($U=1.125$, top left), case 2 ($U=0.5$, top right), case 3 ($U=1.25$, bottom left), and case 4 ($U=1.125$, bottom right). In each case, the breakdown of the real (thin dashed lines) and imaginary (thin dotted lines) components of the WKBJ solution (25) about the turning point is observed, while the real (thick dashed lines) and imaginary (thick dotted lines) components of the WKBJ turning point expansion are in much closer agreement with the numerical profiles (thin solid lines). The parameters chosen in each case match the numerical profiles illustrated in Fig. 6, with case 1 (bottom left), case 2 (top left), case 3 (bottom right), and case 4 (bottom left).

Of the four cases to be described, the agreement between the WKBJ turning-point solution and the numerical solution is least good in case 1, where the imaginary parts of the composite turning-point solution and the numerical solution show a relative error of 26% at the turning point. This is because the local turning-point solution $W_0$ in (28b) has to be employed to describe the behaviour close to the wall, the turning point, and near the centreline. It is expedient to investigate this case even if it is less accurate than the subsequent cases, because the coefficients (46) are also present in the more complicated expansions. For cases 2, 3, and 4, the composite expansions are in excellent agreement with the numerical solutions.

## Appendix D: Errors in numerical and asymptotic solutions

In order to assess the relative accuracy of the asymptotic approximations, the error measure

$$\begin{aligned} E = \max _{0 \le y \le 1} \bigl |\left| W \right| - \left| W_\text {num} \right| \bigr |, \end{aligned}$$

is calculated, where *W* is the approximate solution generated by each approach and $W_\text {num}$ is the numerical solution calculated utilizing the method outlined in Sect. 4. Profiles are compared on a uniform mesh with 8000 uniformly spaced points over the range $0 \le y \le 1$. In addition to determining the errors associated with each asymptotic method, the error associated with the numerical solution is estimated via a comparison with a second numerical solution calculated on a grid with a mesh spacing of exactly double that of the first numerical solution. Figure 14(left) shows the error in the absence of a turning point ($U=10$) for the numerical solution (thick solid line), the boundary layer approximation (dashed line), and the WKBJ approximation (dotted line) for variations of $\varepsilon$.

For the range of values of $\varepsilon$ investigated:

- The error in the numerical solution increases as the value of $\varepsilon$ decreases. This is because smaller values of $\varepsilon$ produce higher wall-normal velocity gradients close to the channel wall and, as $\varepsilon$ decreases, the approximation of these gradients on a uniform grid becomes less accurate.
- For $\varepsilon \gtrsim 10^{-2}$, the WKBJ method outperforms the boundary-layer approximation. In this region the errors associated with the two methods decay at comparable rates as the value of $\varepsilon$ decreases. The decay of *E* as $\varepsilon$ decreases is fully expected as $\varepsilon$ is the small asymptotic parameter.
- For $\varepsilon \lesssim 10^{-2}$, the apparent errors associated with the asymptotic solutions start to increase as the value of $\varepsilon$ decreases. This is because the asymptotic solutions are compared with the numerical solution. The asymptotic solutions tend to the exact solution as $\varepsilon \rightarrow 0$, whereas the numerical error increases, as discussed in the first bullet point.
- For very small $\varepsilon$, the errors associated with the WKBJ method and the boundary-layer method are very similar, which suggests that the two asymptotic methods are in close agreement both with each other and with the exact solution. This is to be expected for small $\varepsilon$.

To investigate the relative accuracy of the WKBJ turning-point expansion and the Langer method when a turning point is present, the error associated with each method is calculated for $U=1.125$ ($y_0=0.5$) and a range of values of $\varepsilon$, with the errors shown in Fig. 14(right). In terms of the hierarchy of flow solutions with turning points illustrated in Fig. 12(right), the solution about the turning point is valid across the full half channel for $\varepsilon \gg 2^{-5}$ (Sect. 6.1, case 1), while the full five-term composite expansion (27) is valid for $\varepsilon \ll 2^{-3}$ (Sect. 6.1, case 4). The limits of validity for case 1 and case 4 are denoted by vertical lines in Fig. 14(right).

- The Langer method outperforms the turning-point expansion (case 4) across a wide range of values of $\varepsilon$. The decay rate of both methods is similar as the value of $\varepsilon$ decreases.
- Throughout the region of common validity ($2^{-5} \ll \varepsilon \ll 2^{-3}$), the error associated with the five-term composite expansion (case 4) is smaller than that observed with just the turning-point solution (case 1). This is consistent with the behaviour shown for $\varepsilon =0.05$ in Sect. 6.1.
- For $\varepsilon \lesssim 10^{-2}$, the asymptotic methods are more accurate than the numerical method, as shown in Fig. 14.
- For very small $\varepsilon$, good agreement between the turning point expansion and the Langer solution is obtained as both tend to the exact solution.
- For $\varepsilon \approx 1$, the errors associated with the turning point expansion and the Langer method are $O\,(1)$, and hence these asymptotic methods are of no use in this regime. This is in marked contrast to the earlier analysis for $U=10$, where the errors associated with the boundary-layer method ($E=4.0\times 10^{-3}$) and the WKBJ approximation ($E=2.4\times 10^{-4}$), when $\varepsilon = 1$ indicate that these methods may still be of use.
